# Tropical Peatland Hydrology Simulated With a Global Land Surface Model

**DOI:** 10.1029/2021MS002784

**Published:** 2022-02-28

**Authors:** S. Apers, G. J. M. De Lannoy, A. J. Baird, A. R. Cobb, G. C. Dargie, J. del Aguila Pasquel, A. Gruber, A. Hastie, H. Hidayat, T. Hirano, A. M. Hoyt, A. J. Jovani‐Sancho, A. Katimon, A. Kurnain, R. D. Koster, M. Lampela, S. P. P. Mahanama, L. Melling, S. E. Page, R. H. Reichle, M. Taufik, J. Vanderborght, M. Bechtold

**Affiliations:** ^1^ Department of Earth and Environmental Sciences KU Leuven Heverlee Belgium; ^2^ School of Geography University of Leeds Leeds UK; ^3^ Center for Environmental Sensing and Modeling Singapore‐MIT Alliance for Research and Technology Singapore Singapore; ^4^ Instituto de Investigaciones de la Amazonia Peruana (IIAP) Iquitos Peru; ^5^ Universidad Nacional de la Amazonia Peruana (UNAP) Iquitos Peru; ^6^ School of GeoSciences University of Edinburgh Edinburgh UK; ^7^ Research Center for Limnology National Research and Innovation Agency Cibinong Indonesia; ^8^ Research Faculty of Agriculture Hokkaido University Sapporo Japan; ^9^ Department of Earth System Science Stanford University Stanford CA USA; ^10^ UK Centre for Ecology and Hydrology Bangor UK; ^11^ School of Biosciences University of Nottingham Loughborough UK; ^12^ Faculty of Chemical Engineering Technology Universiti Malaysia Perlis Kangar Malaysia; ^13^ Department of Soil Science Lambung Mangkurat University Banjarmasin Indonesia; ^14^ Global Modeling and Assimilation Office NASA Goddard Space Flight Center Greenbelt MD USA; ^15^ Department of Forest Sciences University of Helsinki Helsinki Finland; ^16^ Science Systems and Applications Inc. Lanham MD USA; ^17^ Sarawak Tropical Peat Research Institute Kuching Malaysia; ^18^ School of Geography, Geology and the Environment University of Leicester Leicester UK; ^19^ Department of Geophysics and Meteorology IPB University Bogor Indonesia; ^20^ Agrosphere Institute IBG‐3 Forschungszentrum Jülich Jülich Germany

**Keywords:** PEATCLSM, groundwater, drainage, peat swamp, evaporation, wetland

## Abstract

Tropical peatlands are among the most carbon‐dense ecosystems on Earth, and their water storage dynamics strongly control these carbon stocks. The hydrological functioning of tropical peatlands differs from that of northern peatlands, which has not yet been accounted for in global land surface models (LSMs). Here, we integrated tropical peat‐specific hydrology modules into a global LSM for the first time, by utilizing the peatland‐specific model structure adaptation (PEATCLSM) of the NASA Catchment Land Surface Model (CLSM). We developed literature‐based parameter sets for natural (PEATCLSM_Trop,Nat_) and drained (PEATCLSM_Trop,Drain_) tropical peatlands. Simulations with PEATCLSM_Trop,Nat_ were compared against those with the default CLSM version and the northern version of PEATCLSM (PEATCLSM_North,Nat_) with tropical vegetation input. All simulations were forced with global meteorological reanalysis input data for the major tropical peatland regions in Central and South America, the Congo Basin, and Southeast Asia. The evaluation against a unique and extensive data set of in situ water level and eddy covariance‐derived evapotranspiration showed an overall improvement in bias and correlation compared to the default CLSM version. Over Southeast Asia, an additional simulation with PEATCLSM_Trop,Drain_ was run to address the large fraction of drained tropical peatlands in this region. PEATCLSM_Trop,Drain_ outperformed CLSM, PEATCLSM_North,Nat_, and PEATCLSM_Trop,Nat_ over drained sites. Despite the overall improvements of PEATCLSM_Trop,Nat_ over CLSM, there are strong differences in performance between the three study regions. We attribute these performance differences to regional differences in accuracy of meteorological forcing data, and differences in peatland hydrologic response that are not yet captured by our model.

## Key Points

- For the first time, a global land surface model was adapted to include natural and drained tropical peatland hydrology
- Evaluation with in situ data shows that the tropical natural and drained peatland model versions both outperform the default model version
- Regional skill differences are attributed to accuracy differences of model parameterization and meteorological forcing data across regions

## 1 Introduction

Peatlands are wetlands with an organic soil surface layer, i.e., peat. Their waterlogged, anoxic environment greatly reduces the decomposition of plant litter, facilitating the accumulation of a carbon‐rich layer that can be up to several meters deep. Peatlands cover about 3% of the Earth's land surface (Leifeld & Menichetti, 2018; Xu et al., 2018; Yu et al., 2010), but make up about 25% of the global soil carbon (C) pool (Scharlemann et al., 2014; Yu et al., 2010). External disturbances such as climate change, land use change or drainage put these immense, long‐term C stocks at risk of becoming strong greenhouse gas sources.

Despite long denial of their possible existence (Joosten, 2016), tropical peatlands are now estimated to constitute about 13% of the global peatland area (Leifeld & Menichetti, 2018). They are predominantly located in low‐altitude areas of Central and South America, Africa, and Southeast Asia, although some high‐altitude peatlands occur in the mountain ranges of Africa, South America (Chimner et al., 2019) and Papua New Guinea (Page, Rieley, & Banks, 2011). Despite many research efforts to map peatlands globally (Dargie et al., 2017; Draper et al., 2014; Gumbricht et al., 2017; Leifeld & Menichetti, 2018; Miettinen et al., 2016; Xu et al., 2018), uncertainties in the peatland extent remain. Data on tropical peatlands is limited and often of poor quality, and some peatlands like the Cuvette Centrale peatland complex in the Congo Basin (Dargie et al., 2017) were only recently described. Comparison of the estimated C storage in various biomes suggests that tropical peatlands are among the most C‐dense terrestrial ecosystems on Earth (Joosten & Couwenberg, 2008): upland forests in the Amazon Basin store about 250–300 Mg C ha^−1^ (split about equally above‐ and belowground; Coronado et al., 2021; Draper et al., 2014), boreal peatlands store about 1,350 Mg C ha^−1^ (Yu et al., 2010), and, depending on the peatland type, tropical peatlands store between 685 (41 aboveground: 644 belowground) Mg C ha^−1^ and 1,752 (108 aboveground: 1,644 belowground) Mg C ha^−1^ (Coronado et al., 2021; Draper et al., 2014; Murdiyarso et al., 2009; Saragi‐Sasmito et al., 2019).

Most well‐studied tropical peatlands are raised bogs (Page et al., 2006), i.e., mostly rain‐fed, ombrotrophic (nutrient‐poor), and dome‐shaped peatlands (Anderson, 1983). The water level of those peatlands conforms to the general dome morphology of the bog and therefore is relatively uniform to the surface (Cobb et al., 2017; Dommain et al., 2010). Lähteenoja et al. (2009) demonstrated the occurrence of both ombrotrophic and minerotrophic swamps in the Peruvian Amazon. Although the peatland types in the Congo Basin are poorly mapped (Dargie et al., 2017), the diverse vegetation and flooding dynamics indicate that ombrotrophic and minerotrophic peatlands likely exist together. Periodic flooding with nutrient‐rich water from rivers or lakes, and/or lateral surface water discharge is typical for minerotrophic peatlands but may also occur in largely ombrotrophic peatlands.

The seasonal dynamics of the water level (negative below the surface) are mainly determined by the balance between precipitation (*P*), as main water input in ombrotrophic peatlands, and five major water loss pathways: evaporation from canopy interception, evaporation from soil and free‐standing water, plant stomatal transpiration, overland flow, and water flow through the peat soil (Baird et al., 2017; Mezbahuddin et al., 2015). During the wet season, *P* often exceeds evapotranspiration (ET) and leads to high (=shallow) water levels that can reach above the peatland surface. This ground surface is characterized by microforms—elevated surface areas or hummocks and depressions or hollows—that affect the lateral discharge (*Q*). Lateral hydraulic gradients are generally low over the scale of the peat dome, but surface inundation results in large lateral water flow rates across the flooded fraction of the peatland surface (overland flow) and through the top layer of the peat (subsurface runoff) simultaneously. In periods with low *P*, the water level recedes, flooding diminishes and the *Q* decreases, eventually limiting further water level drawdown (Dommain et al., 2010; Mezbahuddin et al., 2015).

The improved understanding of tropical peatland hydrology and the peat‐specific features that regulate it has led to the development of small‐scale hydrology models for both natural (Baird et al., 2017; Cobb et al., 2017; Wösten et al., 2008) and drained (Baird et al., 2017; Mezbahuddin et al., 2015; Wösten et al., 2008) tropical peatlands. The seasonal and interannual water level variations of and differences between natural and drained tropical peatlands has been studied over a range of small scales, i.e., from the hummock‐hollow scale (Mezbahuddin et al., 2015) to regional groundwater flow (Ishii et al., 2016; Wösten et al., 2008).

Artificial drainage consistently lowers the water level throughout the year (Hirano et al., 2015; Taufik et al., 2020) and can result in very low (=deep) water levels of up to 2 m below the surface in the dry season. Inadequate vertical water recharge exposes the peat soil to drying, leading to irreversible lowering of peat layers through subsidence (Evans et al., 2019; Hooijer et al., 2012; Mezbahuddin et al., 2015; Young et al., 2017), large C losses through rapid biological oxidation, increased peat bulk density (Hooijer et al., 2012), and an increased vulnerability to wildfires (Page et al., 2002; Taufik et al., 2017; Turetsky et al., 2015). Hoyt et al. (2020) estimated that over 90% of Southeast Asian peatlands are subsiding at an average rate of 2.24 cm yr^−1^, which translates into an annual C loss of 155 Mt C yr^−1^. All (northern, temperate and tropical) drained peatlands together emit nearly 5% of the global anthropogenic CO_2_ emissions, even though they cover only 0.4% of the Earth's land area (Joosten, 2015). Recent studies by Leifeld and Menichetti (2018), Leifeld et al. (2019), and Günther et al. (2020) illustrated that peatland restoration, of tropical peatlands in particular, is possibly one of the most efficient ways of global climate change mitigation. However, the success of restoring or rehabilitating degraded peatlands and conserving intact peatlands strongly depends on a proper understanding of peatland hydrology and water regimes (Evans et al., 2021; Murdiyarso et al., 2019).

State‐of‐the‐art Earth system models, which are used for future climate projections, currently do not include peatland ecosystems (Loisel et al., 2021). However, the need to more accurately monitor and predict greenhouse gas emissions has pushed the development of complex biogeochemical modules for simulating carbon and nitrogen cycling in ecosystem and Earth system models. These biogeochemical modules depend on a proper representation of peat‐specific hydrology, which is difficult to parameterize at large scales (Limpens et al., 2008) and therefore often inadequately accounted for in global Earth system models.

Land Surface Models (LSMs) can provide land energy and water fluxes for these Earth system models, and recently some peat‐specific hydrology modules have been developed for different LSMs such as the Canadian Land Surface Scheme (CLASS; Wu et al., 2016), the Lund‐Potsdam‐Jena (LPJ) model (Wania et al., 2009), the Community Land Model (CLM; Shi et al., 2015), the Organizing Carbon and Hydrology In Dynamic Ecosystems (ORCHIDEE; Qiu et al., 2018) LSM, and the Catchment Land Surface Model (CLSM; Bechtold et al., 2019). The CLASS and LPJ models modified their soil layering to better represent the depth‐specific peat properties. Next to the humification‐based soil layering that was already included in CLASS, Wu et al. (2016) added a moss layer that buffers the soil water and energy exchange, whereas Wania et al. (2009) integrated an acrotelm‐catotelm structure to the layering of the LPJ model. Both models did not consider the influence of peatland microtopography on the hydrology of peatlands, in contrast to Shi et al. (2015) who integrated the effect of microtopography to simulate a dynamic water level in CLM. In the peat‐specific hydrology module in ORCHIDEE, all surface runoff from the non‐peatland fraction of a grid cell was used as additional water input into the peatland fraction of that grid cell, mimicking the hydrological situation of groundwater and surface water influence in minerotrophic (fens) and not of ombrotrophic (bogs) peatlands (Qiu et al., 2018). CLSM (Koster et al., 2000) is the land model component of the NASA Goddard Earth Observing System (GEOS) modeling framework and is used for operational purposes. CLSM is one of the few global LSMs that simulates a dynamic water level, and Bechtold et al. (2019) used the CLSM framework to model the effect of microtopography on the water level, among other peat‐specific parameterizations, to represent bogs in their peat‐specific module (PEATCLSM). However, the above peat modules focus on natural northern peatlands only. Despite many similarities between tropical and northern peatlands, distinct structural and physical characteristics result in different hydrological dynamics.

**Figure 1:**
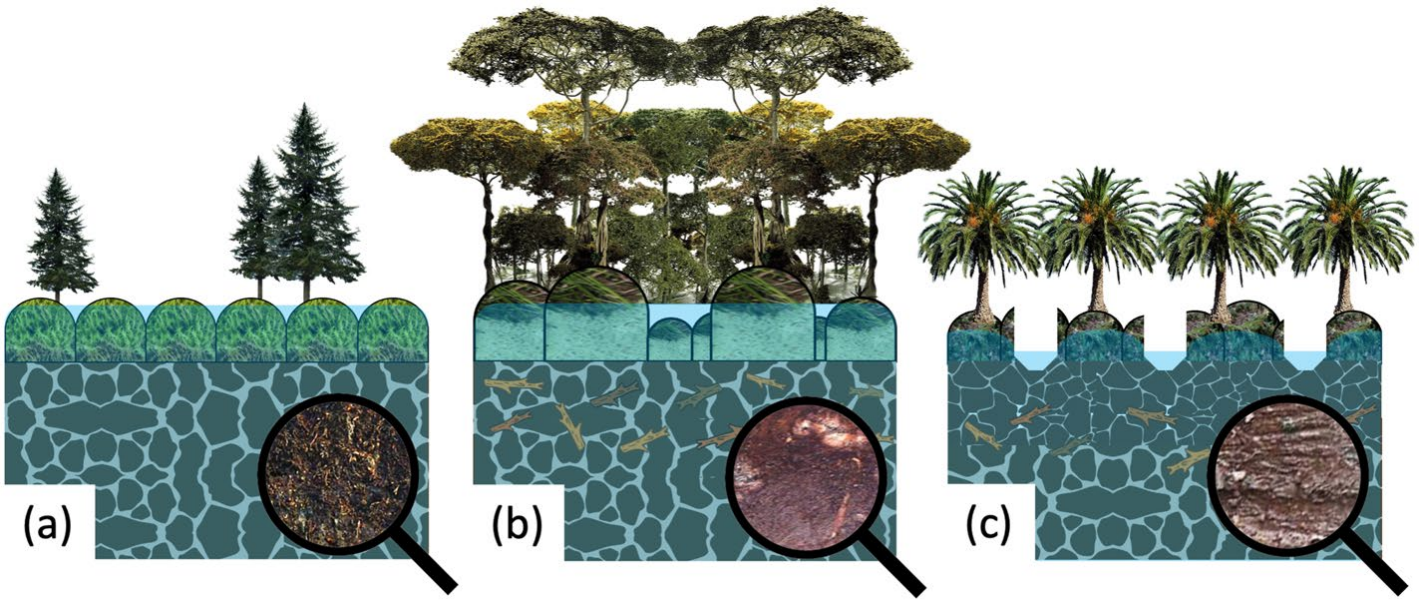
The structural and physical differences (discussed in the text) between (a) natural northern, (b) natural tropical, and (c) drained tropical peatlands that are relevant from a land surface modeling perspective, and result in distinct hydrological dynamics. The magnifying glasses depict a close‐up of a (a) natural northern peat soil, (b) natural tropical peat soil with woody remains, and (c) drained and compacted tropical peat soil.

Figure 1 shows some of the main differences between natural northern, natural tropical, and drained tropical peatlands from a land surface modeling perspective. Northern peatlands are often dominated by bryophytes (such as *Sphagnum* mosses) with sparse vascular vegetation (such as coniferous trees, shrubs, and sedges), whereas natural tropical peat swamp forests often have a multilayered, dense canopy with a variety of trees (hardwood or palm), and drained tropical peatlands are often covered with industrial plantations of oil palm (*Elaeis guineensis*; the source of palm oil) or *Acacia* species (source of pulpwood), small‐holder agriculture, and shrubs and ferns (Miettinen et al., 2016). Northern peatlands often have a regular and perpendicular oriented microtopographic pattern that reduces lateral water flow, this pattern has not yet been observed in tropical peatlands (Lampela et al., 2016). Peat drainage strongly reduces the original surface microtopography (Lampela et al., 2017), consistently lowers the water level by increased lateral water flow through drainage canals that incise deeply in the peat, and results in shrinkage (in addition to mechanical compaction) of (mainly) the top 0.5 m of peat (Hooijer et al., 2012).

To our knowledge, there is no global LSM in the peer‐reviewed literature that has been parameterized and evaluated for either natural or drained tropical peatlands. Here, we developed the first, large‐scale hydrological modules for both natural and drained tropical peatlands for use in a global LSM, by utilizing the recent, northern peatland‐specific adaptations of CLSM, i.e., PEATCLSM (Bechtold et al., 2019). We collected the limited data on tropical peatlands available in the literature to construct a set of hydrological model parameters, and a unique data set of water level and eddy covariance‐derived ET for model evaluation over tropical peatlands in Central and South America, the Congo Basin, and Southeast Asia.

In Section 2 we describe the CLSM and PEATCLSM model structures, and how we developed a tropical PEATCLSM module (PEATCLSM_Trop_) for natural (PEATCLSM_Trop,Nat_) and drained (PEATCLSM_Trop,Drain_) tropical peatlands using separate literature‐based parameter sets. Our experimental design and the evaluation methods, including the development of an extensive evaluation data set of water level and ET observations, are also described in Section 2. In Section 3 we show our results and compare them to our evaluation data set. The results are discussed in Section 4, and conclusions on model performance and shortcomings, relevant findings, and future possibilities are presented in Section 5.

## 2 Materials and Methods

### 2.1 Global Land Surface Modeling

#### 2.1.1 Catchment Land Surface Model

CLSM (Ducharne et al., 2000; Koster et al., 2000) is a state‐of‐the‐art LSM that is part of the NASA GEOS global modeling framework. GEOS is used to generate operational global forecast and analysis products (https://gmao.gsfc.nasa.gov/products/), such as the Modern‐Era Retrospective analysis for Research and Applications, Version 2 (MERRA‐2; Bosilovich et al., 2016). The analysis and forecasts serve as background to various satellite retrievals and are also used in the generation of the operational Soil Moisture Active Passive (SMAP) mission Level‐4 Surface and Root‐Zone Soil Moisture (L4_SM) data assimilation product (Reichle et al., 2019). Here, we used the version of CLSM that is used for version 3 of the L4_SM algorithm (Reichle et al., 2019) and includes peat as a soil class following a soil parametrization update by De Lannoy et al. (2014). Vereecken et al. (2019) compares the different components of CLSM to other LSMs, and Bechtold et al. (2019) gives a more detailed description of the CLSM components that were used for the development of northern peatland hydrology in PEATCLSM.

**Figure 2:**
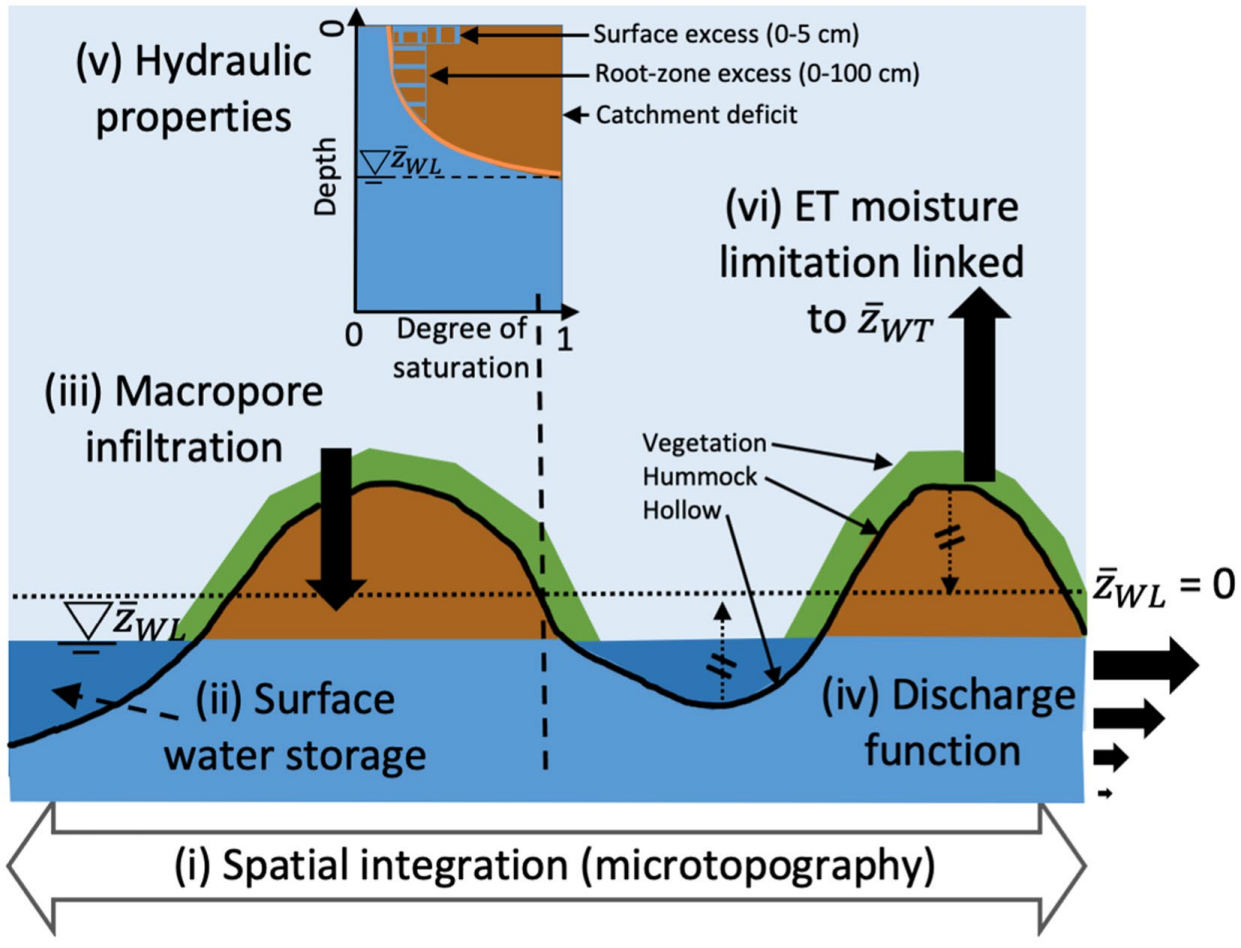
Schematic illustration of the six (i–vi, discussed in the text) peatland‐specific adaptations and parameter updates implemented in PEATCLSM (adapted from Bechtold et al. [2020]). ${\bar{z}}_{WL}$ is the grid cell mean water level.

CLSM uses the distribution of the topographic index (TOPMODEL approach; Beven & Kirkby, 1979) within the computational land surface element to estimate the spatial distribution of surface (0–5 cm) soil moisture (*θ*_5cm_), root‐zone (0–100 cm) soil moisture, and dynamic water level (${\bar{z}}_{WL}$; negative downwards). CLSM is one of the few global LSMs that simulates a ${\bar{z}}_{WL}$ (Vereecken et al., 2019), with the overbar implying that it is a grid cell average of the subgrid variability in water level. These diagnostic soil moisture and groundwater variables are computed from three model prognostic variables (Figure 2):

1. Catchment deficit (surface to bedrock): is defined as the amount of water per unit area that would be needed to saturate the soil of the entire catchment for a given ${\bar{z}}_{WL}$, assuming an initial hydrostatic equilibrium profile.
2. Root‐zone excess (0–100 cm): the moisture disequilibrium (due to input or extraction of water) from the assumed hydrostatic equilibrium profile in the top 100 cm.
3. Surface excess (0–5 cm): the moisture disequilibrium in the top 5 cm from the equilibrium moisture profile as modified by the root‐zone excess.

Vertical water flow between the surface and root‐zone excess, and between the root‐zone excess and the catchment deficit is controlled by two timescale parameters. The empirical equations for these timescale parameters (Ducharne et al., 2000) were fitted (prior to LSM simulation) to offline Richards equation simulations. To solve the Richards equation, sets of prognostic variables were combined with a soil‐specific Campbell parameterization (see Section 2.2.3; Campbell, 1974) over a high‐resolution, vertical soil column:

(1) $$\frac{h}{h_{S}}={\left(\frac{\theta }{\theta_{S}}\right)}^{-b}$$

(2) $$K=K_{S}{\left(\frac{\theta }{\theta_{S}}\right)}^{2b+3}$$

where *h* is the pressure head (cm H_2_O), *h*_S_ is the air entry pressure (cm H_2_O), *θ* is the volumetric soil moisture content (m^3^ m^−3^), *θ*_*S*_ is the volumetric soil moisture content at saturation (m^3^ m^−3^), *b* is an empirical shape parameter (−), K is the unsaturated hydraulic conductivity (m s^−1^), and K_S_ is the saturated hydraulic conductivity (m s^−1^).

At each model timestep, the spatial land surface element is partitioned into three areal fractions (*F*) with distinct hydrological regimes: the saturated region (*F*_sat_), the unsaturated‐but‐transpiring fraction (*F*_tra_), and the wilting fraction (*F*_wilt_), with *F*_sat_ + *F*_tra_ + *F*_wilt_ = 1 (Bechtold et al., 2019; Koster et al., 2000). These fractions are obtained by shifting the distribution of equilibrium root‐zone moisture (i.e., that is tied to the catchment deficit and the associated distribution of ${\bar{z}}_{WL}$) toward drier or wetter conditions based on the root‐zone excess.

#### 2.1.2 Original PEATCLSM Module

The TOPMODEL approach used in CLSM is not optimal for peatlands because most of them are virtually flat on a macrotopographic scale of kilometers, and bogs (and to a lesser extent fens) appear hydraulically decoupled from the groundwater hydrology of the rest of the catchment (Bechtold et al., 2019, 2020). This decoupling is either due to impermeable sediments at the peat base or due to accumulated peat that lifted the peat surface (and water level) above the range of the groundwater fluctuations in the underlying aquifer. Bechtold et al. (2019) replaced the TOPMODEL approach with a peatland‐specific module for natural northern peatlands, from here onwards referred to as PEATCLSM_North,Nat_, of which the fundamental adaptations are shown in Figure 2. Instead of computing the effect of catchment‐scale topography on subsurface hydrology, Figure 2 shows that the microtopography was used to (i) modulate water storage dynamics through regulation of the spatially variable thickness of the unsaturated zone (Dettmann & Bechtold, 2016), and to (ii) allow water ponding in hollows, above the saturated soil. (iii) The large fraction of macropores in the peat surface layers was represented with a very high saturated hydraulic conductivity (K_S,macro_) that resulted in (iv) a *Q* function that non‐linearly declines over the first tens of centimeters of the peat soil. These model changes turned off both the Hortonian (*P* rate > maximum infiltration capacity) and Dunne (saturation excess) overland flow mechanisms. The macropore fraction allowed any *P* on the unsaturated surfaces to infiltrate, while *P* on the flooded hollows (saturated soil) was retained by the unsaturated hummocks and was thus not removed as overland flow. In short, all *P* throughfall eventually leads to water level changes that in turn controls *Q* via the non‐linear discharge function. Furthermore, a peat‐specific revision of (v) the peat matrix hydraulic properties and (vi) a stress function that linked the ET reduction during droughts to the variable water level were also included. In general, PEATCLSM_North,Nat_ simulated higher and spatially less variable water levels, and less ET compared to CLSM, resulting in a significantly better agreement with in situ observations (Bechtold et al., 2019).

All functions and parameters of PEATCLSM_North,Nat_ were constrained with literature data, without any parameter tuning. The same approach was kept in the development of the tropical versions of PEATCLSM, i.e., PEATCLSM_Trop,Nat_ and PEATCLSM_Trop,Drain_, to allow a possible integration of PEATCLSM_Trop_ in GEOS for operational global applications.

### 2.2 Tropical Version of the PEATCLSM Module

#### 2.2.1 Natural and Drained Tropical PEATCLSM Modules

**Figure 3:**
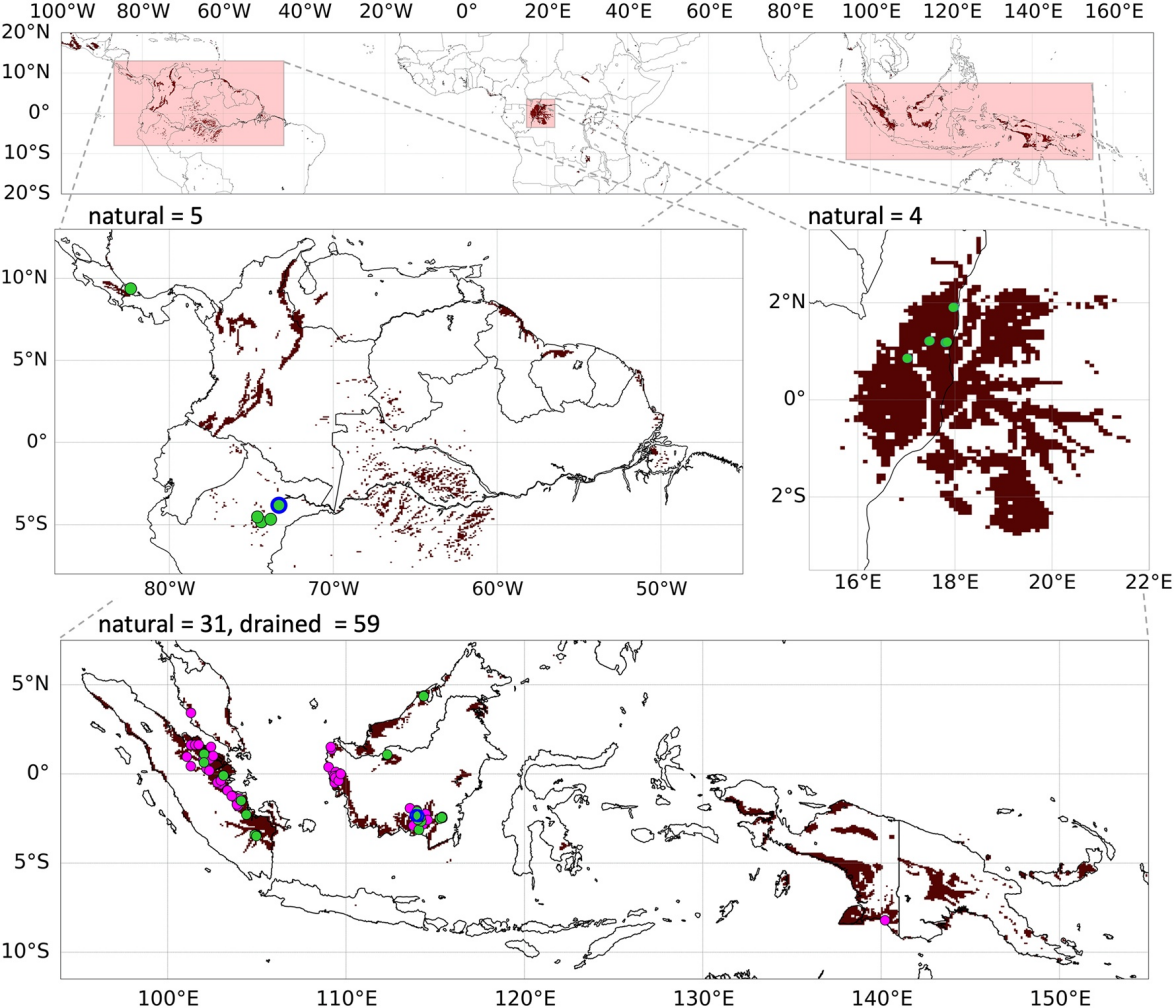
(Top) Distribution of tropical peatlands based on the fusion of PEATMAP (Xu et al., 2018) and the peat distribution used for SMAP L4_SM (De Lannoy et al., 2014). The (brown) peat pixels are projected on the Equal Area Scalable (EASE) grid, version 2.0 (Brodzik et al., 2012) at a spatial resolution of 9 km. (middle and bottom) Three zooms into the major tropical peatland regions of Central and South America, the Congo Basin, and Southeast Asia; also shown are the locations of sites with in situ water level data in (green) natural and (pink) drained peatlands. Sites with in situ eddy covariance data are marked with a blue edge.

The spatial distribution of tropical peatlands is shown in Figure 3. Most well‐studied tropical peatlands are natural ombrotrophic lowland peatlands (Page et al., 2006) but other tropical peatland types (e.g., minerotrophic or highland) occur too. Because of insufficient information to differentiate between tropical peatland types, an “average” parameter set for tropical ombrotrophic lowland peatlands was derived from literature for the PEATCLSM_Trop,Nat_ and PEATCLSM_Trop,Drain_ modules.

Artificial drainage of tropical peatlands, often associated with land cover and land use change, strongly affects the hydrophysical properties of peat soils. Drained peatlands have lower water levels, and the oxic conditions and nitrogen from peat mineralization limits their C accumulation (Leifeld et al., 2020), leading to: reduction of macropores, increased bulk density, reduced saturated hydraulic conductivity, lower soil moisture content, and peat subsidence (Anshari et al., 2010; Ghimire et al., 2018; Kurnain, 2018; Tonks et al., 2017). Therefore, two PEATCLSM_Trop_ modules were developed by constructing separate literature‐based “average” parameter sets, one for natural tropical peatlands (i.e., PEATCLSM_Trop,Nat_) and one for drained tropical peatlands (i.e., PEATCLSM_Trop,Drain_). In the following sections, we present the differences in parameter sets and the limited literature data they were derived from. Table 1 summarizes some parameter settings for the different model versions.

#### 2.2.2 Peatland Microtopography

In both PEATCLSM_Trop_ modules, the TOPMODEL approach from CLSM was replaced by a microtopographic distribution to modulate water level dynamics, similar as in PEATCLSM_North,Nat_ for northern peatlands (Bechtold et al., 2019). The microtopography and soil hydraulic properties (see Section 2.2.3) are crucial in determining the specific yields of shallow groundwater systems, both at high water levels (including surface inundation) and low water levels. The effect of the microtopography on the specific yield depends on its interaction with the soil water retention function and can lead to lower as well as higher soil specific yield at certain water levels (Dettmann & Bechtold, 2016).

**Figure 4:**
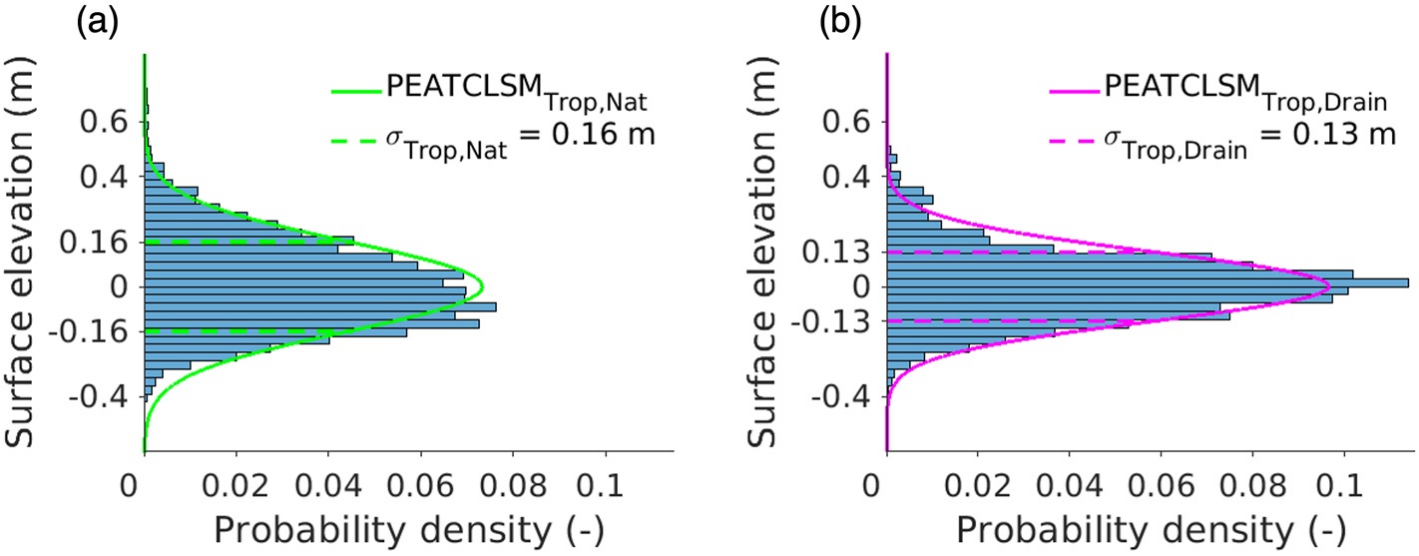
(a) Histogram of the 3,389 surface elevations measured by Lampela et al. (2016) in a natural tropical peatland, together with the derived zero‐mean normal distribution (solid line) and corresponding standard deviation (*σ* = 0.16 m; dashed lines) and (b) histogram of the 3,720 surface elevations measured by Lampela et al. (2017) in a drained tropical peatland, together with the derived zero‐mean normal distribution (solid line) and corresponding standard deviation (*σ* = 0.13 m; dashed lines).

For natural peatlands, Lampela et al. (2016) reported the only available extensively measured surface elevations (3,389 measurements) along a transect in the Sebangau forest (2°32′S, 113°90′E). These surface elevation data were used to construct the microtopographic distribution for PEATCLSM_Trop,Nat_, shown in Figure 4a. The surface reference of the original data was shifted to the mean surface elevation (Figure 2), so that the surface elevation measurements could be approximated by a zero‐mean normal distribution with a standard deviation of 0.16 m (neglecting the minor skewness; Figure 4a), which is larger than the 0.11 m standard deviation used by Bechtold et al. (2019) for PEATCLSM_North,Nat_. Despite the limited geographical area and specific land cover of the surface elevation measurements, the distribution in Figure 4a is consistent with sporadically measured surface elevations in natural tropical peatlands in Southeast Asia or South America (Dommain et al., 2010; Freund et al., 2018; Kelly et al., 2014; Page, Morrison, et al., 2011; Shimamura & Momose, 2007; Swindles et al., 2014). Quantitative data on microtopography from natural tropical peatlands in the Congo Basin remain unavailable, but a few in‐field descriptions indicate that the microtopographic distribution in Figure 4a is likely a good approximation for that region.

Drainage, or degradation more generally, of natural tropical peatlands strongly reduces the original surface microtopography that was developed through a dynamic interaction between vegetation and peat hydrology (Dommain et al., 2010; Jauhiainen et al., 2008; Lampela et al., 2016). The reduction in the microtopography range is often due to the loss of the highest hummock formations. However, some characteristic microforms remain because of uneven subsidence and small burn scars (Ballhorn et al., 2009; Dommain et al., 2010; Lampela et al., 2016). Lampela et al. (2017) observed a flat surface topography with sparse depressions and measured 3,720 surface elevations that were used to derive a microtopographic distribution for PEATCLSM_Trop,Drain_, shown in Figure 4b. The mean surface elevation was calculated and used as the surface reference, in a similar way to that used for PEATCLSM_Trop,Nat_. Figure 4b shows that the measurements could be approximated by a zero‐mean normal distribution with a standard deviation of 0.13 m. This microtopographic distribution is in line with the range of 0.3–0.5 m between the hummocks and hollows observed by Jauhiainen et al. (2008) in two degraded (logged, burned, and drained) tropical peatlands.

#### 2.2.3 Peat Hydraulic Properties: Matrix and Macropores

**Figure 5:**
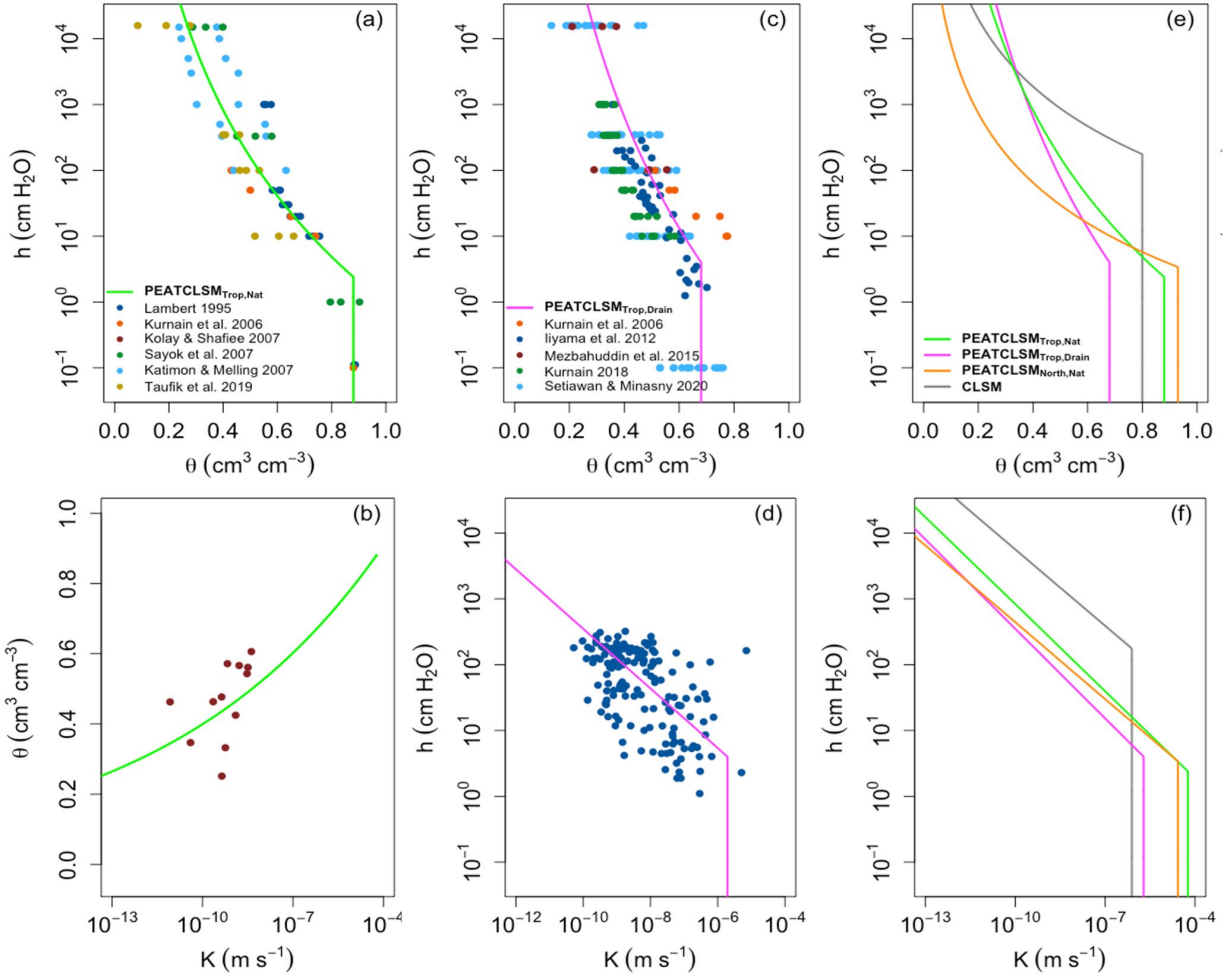
“Average” hydraulic functions for tropical peatlands fitted to multiple literature sources (color‐coded). Retention curve for (a) natural and (c) drained tropical peatlands, and the corresponding unsaturated hydraulic conductivity curve for (b) natural and (d) drained tropical peatlands. Comparison of the (e) soil moisture retention and (f) unsaturated hydraulic conductivity functions for PEATCLSM_Trop,Nat_ (green) and PEATCLSM_Trop,Drain_ (pink) to those from CLSM (gray; De Lannoy et al., 2014) and PEATCLSM_North,Nat_ (orange; Bechtold et al., 2019). Note the different axes for (b) because no K(*h*) data was available for natural tropical peatlands.

The soil hydraulic properties of peatlands vary with depth, and are affected by the degree of humification that is strongly determined by the long‐term water level conditions (Kurnain, 2018). Soil hydraulic input parameters of the peat matrix for PEATCLSM_Trop_ (Table 1) were derived by simultaneously fitting the “average” soil moisture retention and unsaturated hydraulic conductivity functions (Equations 1 and 2) for both natural and drained tropical peatlands, shown in Figure 5. A humification‐based separation (fibric, hemic, and sapric) of the soil hydraulic input parameters was not possible because of a too large within‐class variability.

As opposed to northern peatlands, there is no generally established parameterization of hydraulic functions for the peat matrix of tropical peatlands (Kurnianto et al., 2019; Taufik et al., 2019). Instead, we collected measurements from six literature sources to determine the “average” hydraulic functions for natural tropical peatlands. Five literature sources (Katimon & Melling, 2007; Kurnain et al., 2006; Lambert, 1995; Sayok et al., 2007; Taufik et al., 2019) measured *θ* against *h*, and one (Kolay & Shafiee, 2007) measured K against *θ*. The *θ*_*S*_ of 0.88 cm^3^ cm^−3^ (Table 1) was based on measurements by Lambert (1995), Kurnain et al. (2006), and Sayok et al. (2007). Figure 5a shows that the “average” soil moisture retention function of PEATCLSM_Trop,Nat_ was fitted to data with a large variability, and that the “average” unsaturated hydraulic conductivity function of PEATCLSM_Trop,Nat_ was fitted against *θ* measurements (Kolay & Shafiee, 2007) because no literature data of K against *h* was available. The resulting soil hydraulic input parameters of the peat matrix for PEATCLSM_Trop,Nat_ are shown in Table 1 and were applied in the offline Richards equation simulations (see Section 2.1.1) to obtain the timescale parameters for vertical moisture transfer under unsaturated conditions. The K_S_ of 6 × 10^−5^ m s^−1^ for PEATCLSM_Trop,Nat_ (Table 1) was based on the K_S_ (at a water level of −0.29 m) that Cobb and Harvey (2019) derived from their water level rise and recession curves.

Northern natural peatlands are often described as a two‐layered soil profile that consists of a highly porous, weakly decomposed acrotelm and a more compact catotelm layer (Dettmann et al., 2014; Dimitrov et al., 2010). This structural transition results in a steep gradient in K_S_ from the acrotelm to the catotelm (Hogan et al., 2006; Morris et al., 2015). The structure of peat in natural tropical peatlands is not well characterized; however, a very large K_S_ for the upper peat layers and a much smaller one for the deeper peat layers is established (Baird et al., 2017; Cobb & Harvey, 2019; Kelly et al., 2014).

Artificial drainage results in reduced K_S_ and lower *θ*_*S*_ due to altered peat properties (Ghimire et al., 2018; Kurnain, 2018; Taufik et al., 2019; Tonks et al., 2017), especially in the top layers. To determine the “average” hydraulic functions for drained tropical peatlands, five literature sources were used (Iiyama et al., 2012; Kurnain, 2018; Kurnain et al., 2006; Mezbahuddin et al., 2015; Setiawan et al., 2020). All sources presented *θ* against *h* (Figure 5c), but only Iiyama et al. (2012) measured K against *h* (Figure 5d). Table 1 shows the soil hydraulic input parameters of the peat matrix for PEATCLSM_Trop,Drain_, the *θ*_*S*_ of 0.68 cm^3^ cm^−3^ was based on values from Iiyama et al. (2012), Mezbahuddin et al. (2015), Ghimire et al. (2018), and Kurnianto et al. (2019). The K_S_ of 2 × 10^−6^ m s^−1^ for PEATCLSM_Trop,Drain_ was based on the measurements by Iiyama et al. (2012) (Figure 5d), and is in the range of K_S_ values mentioned by Kurnianto et al. (2019).

Furthermore, the timescale parameter that regulates the moisture transfer between catchment deficit and root‐zone excess (upwards and downwards) was adjusted for PEATCLSM_Trop,Drain_. The initial timescale parameter guess, derived from the offline Richards equation simulations, was representative for the compacted, upper layers of drained tropical peatlands (upper ± 0.5 m), but not for the deeper, less compacted catotelm (Hooijer et al., 2012). Preliminary simulations with this initial guess showed a too long lag in the water level rise at the end of the dry season. Insufficient upward moisture transfer from the catchment deficit during the dry season led to a strong disequilibrium in the unsaturated soil profile, or more specifically, it led to the accumulation of a large negative root‐zone excess (see Section 2.1.1). By contrast, the in situ observed data did show an instant rise of the water level with *P* at the end of the dry season, suggesting no such disequilibrium but a strong vertical coupling between the water level and root zone for deeper peat layers. Therefore, the timescale parameter was given an arbitrary large value that allows a strong coupling of the catchment deficit and the root‐zone excess.

#### 2.2.4 Peatland Discharge

The *Q* in natural tropical peatlands is low for lower water levels and increases non‐linearly following a power law function with rising water levels (Equation 3), becoming very large when water breaches the surface in hollows because this generates surface and subsurface runoff simultaneously. Bechtold et al. (2019) used the empirical, single power function by K. E. Ivanov (given in Romanov [1968]) to describe the *Q* in natural northern peatlands. Since natural tropical peatlands behave similarly, this function was also used to describe the *Q* $\left({\bar{z}}_{WL}\right)$ relation for PEATCLSM_Trop,Nat_:

(3) $$T_{a}\left({\bar{z}}_{WL}\right)=\frac{K_{S,\text{macro},z=0}{\left(1-100{\bar{z}}_{WL}\right)}^{1-\alpha }}{100\left(\alpha -1\right)},\;\mathrm{for}\;\;\alpha >1,\;{\bar{z}}_{WL}\leq 0$$

(4) $$Q\left({\bar{z}}_{WL}\right)=cT_{a}\left({\bar{z}}_{WL}\right)$$

where *T*_a_ is the transmissivity (m^2^ s^−1^), ${\bar{z}}_{WL}$ is the mean grid cell water level (m), K_S,macro,z = 0_ is K_S,macro_ at the mean surface elevation (m s^−1^), *α* is an empirical parameter that describes the rate of K_S,macro_ decrease with depth (−), *Q* $\left({\bar{z}}_{WL}\right)$ is the water level‐dependent discharge (m s^−1^), and *c* is the average hydraulic gradient divided by the average length of the peatland acrotelm in horizontal flow direction (m^−1^).

**Figure 6:**
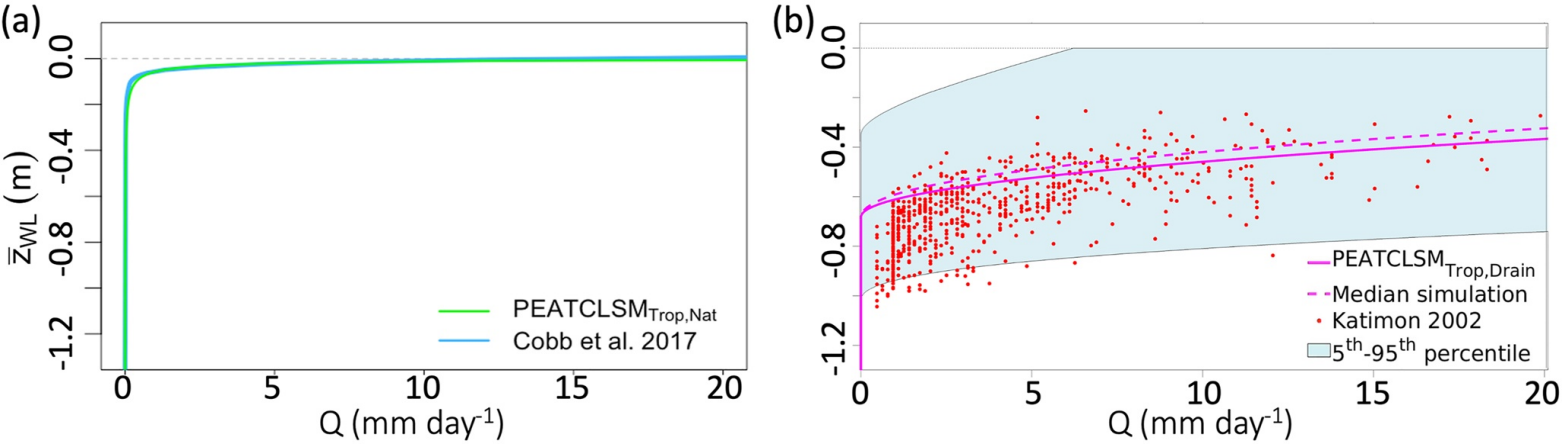
(a) The PEATCLSM_Trop,Nat_ discharge function (green; mm day^−1^) obtained by fitting the function of K. E. Ivanov (given in Romanov [1968]) to the discharge function of Cobb et al. (2017) (blue; indistinguishable from fit). (b) The PEATCLSM_Trop,Drain_ discharge function (solid line; mm day^−1^) and its 95% CI obtained by a Monte Carlo simulation with distributions of the Dupuit‐Forchheimer parameters. The PEATCLSM_Trop,Drain_ discharge function was compared against the median Monte Carlo simulation (dashed line), and 3‐day averaged in situ Q$\left({\bar{z}}_{WL}\right)$ data from Katimon (2002).

CLSM poorly represents the dual hydraulic dynamics of a peat soil (acrotelm and catotelm), and therefore Bechtold et al. (2019) included a K_S,macro_ (m s^−1^) parameter for the high macropore flow rates in the acrotelm for PEATCLSM_North,Nat_, alongside the K_S_ (Section 2.2.3) that represents flow in the catotelm. Despite the absence of a clear acrotelm‐catotelm structure in tropical peatlands, similar high macropore flow rates are observed in the upper soil layers of tropical peatlands. The K_S,macro_ parameter is a peat property but also includes overland flow in hollows, which makes it a property of the entire peatland system rather than just a peat soil property. Cobb and Harvey (2019) reported an estimated K_S,macro_ of 73 m s^−1^ (6.3 × 10^6^ m day^−1^) at 0.17 m above the base of the hollows, which, based on our microtopographic standard deviation for natural peatlands (see Section 2.2.2), almost corresponds to our surface reference (*z* = 0) and thus makes this the K_S,macro,z=0_. However, to fit the Ivanov *Q* function (Equations 3 and 4) to the *Q* function of Cobb and Harvey (2019), a much lower K_S,macro,z=0_ of 7.3 m s^−1^ for PEATCLSM_Trop,Nat_ was used. The *Q* function of Cobb and Harvey (2019) was derived from the specific yield, based on the main rising and recession curves (response of water level to *P* rate), using the Laplacian of the peat surface elevation of a peat dome in Brunei. In PEATCLSM_Trop,Nat_, the Ivanov *Q* function was kept for consistency with PEATCLSM_North,Nat_, but the parameters of the function were fitted to the field‐based *Q* function of Cobb and Harvey (2019). Figure 6a shows both the *Q* function of Cobb and Harvey (2019) and the fitted PEATCLSM_Trop,Nat_ *Q* function (m parameter value of 3), which are almost indistinguishable.

For drained peatlands, the *Q* function of Ivanov is not suitable. In case of drainage, *Q* is strongly influenced by the ditch depth and density (Gong et al., 2012). A water level rise above the bottom of the ditch generates saturated subsurface flow perpendicular to the ditch, where it is efficiently removed by open‐channel flow (Gong et al., 2012; Guertin et al., 1987). Therefore, the Dupuit‐Forchheimer *Q* function for an unconfined aquifer (Gong et al., 2012; Guertin et al., 1987) was used for PEATCLSM_Trop,Drain_ as follows:

(5) $$\begin{array}{cccc} Q\left({\bar{z}}_{WL}\right) & = & 0, & \mathrm{if}\;{\bar{z}}_{WL}\leq z_{ditch}\\ & = & 4K_{\text{S,hrz}}{\left(z_{ditch}-{\bar{z}}_{WL}\right)}^{2}\frac{L_{ditch}}{w_{strip}}, & \mathrm{if}\;0\;m>{\bar{z}}_{WL}>z_{ditch}\\ & = & 4K_{\text{S,hrz}}{\left(z_{ditch}\right)}^{2}\frac{L_{ditch}}{w_{strip}}-\left(\frac{{\bar{z}}_{WL}}{dt}\right), & \mathrm{if}\;{\bar{z}}_{WL}\geq 0\;m \end{array}$$

where *Q* $\left({\bar{z}}_{WL}\right)$ is the water level‐dependent discharge (m day^−1^), ${\bar{z}}_{WL}$ is the mean grid cell water level (m), *z*_ditch_ is the ditch depth (m), K_S,hrz_ is the mean saturated horizontal hydraulic conductivity (m day^−1^), *L*_ditch_ is the total ditch length per drained area (m m^−2^), *w*_strip_ is the ditch interval length (m), and *dt* is the time step (day). The Dupuit‐Forchheimer *Q* function (Equation 5) is well established to describe the discharge of drained peatlands, and its four drainage‐related parameters were set to median values based on literature. K_S,hrz_ was set at 52 m day^−1^ based on Katimon (2002), Firdaus et al. (2010), Firdaus et al. (2012), Ghimire et al. (2018), and Kurnianto et al. (2019). The median parameter value for *L*_ditch_ (= 0.0318 m m^−2^) was based on Dadap et al. (2021), and the mean w_strip_ (= 31.4 m) was based on its inverse relationship to *L*_ditch_. The mean *z*_ditch_ (= −0.68 m) was obtained from measurements in acacia, rubber and oil palm plantations, and intensively logged forests (Biancalani & Avagyan, 2014; Carlson et al., 2015; Evans et al., 2019; Hooijer et al., 2006; Ritzema et al., 1998; Wösten et al., 2008). The average model drainage parameters result in a constant drainage efficiency as is observed in the field, because of regular and sporadic ditch maintenance and deepening by plantation companies and local farmers that keeps pace with peat subsidence.

**Figure A1:**
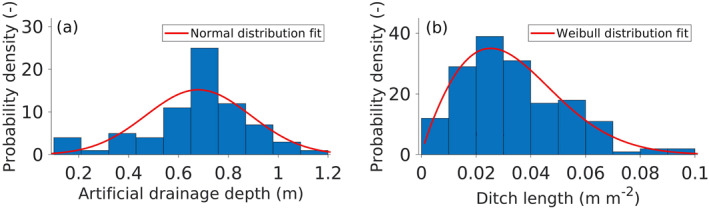
Distributions of two parameters of the Dupuit‐Forchheimer function, (a) ditch depth (*z*_ditch_; in m), and (b) ditch length (*L*_ditch_; in m m^−2^), with their corresponding distribution fit. The ditch interval length (*w*_strip_; in m) is derived from the *L*_ditch_ distribution fit.

To quantify the impact of the parameter variability on *Q*, a Monte Carlo analysis (10^5^ simulations) was performed using distributions for three out of four parameters, as discussed in Figure A1. Figure 6b shows that the median Monte Carlo simulation (dashed line) closely corresponds to the simulation with the median parameter values (solid line). The PEATCLSM_Trop,Drain_ *Q* function (mm day^−1^) is also compared to measurements reported by Katimon (2002). The comparison data are daily *Q* and water level measurements (1986–1994) that were quality checked and, to mitigate measurement noise, averaged with a 3‐day moving window. Most of the comparison data lies within the 95% confidence interval (CI) of the PEATCLSM_Trop,Drain_ *Q* function, although the reported drainage level of −1.60 m allows for much larger *Q* rates at lower water levels (Figure 6b).

#### 2.2.5 Evapotranspiration: Plant Drought and Waterlogging Stress

The nonvascular plants (*Sphagnum* mosses) that often dominate northern peatlands show abrupt drying for a small water level drawdown. The vascular vegetation of tropical peatlands is much less sensitive to a water level drop, and only experiences drought stress at lower water levels. The PEATCLSM_Trop,Nat_ and PEATCLSM_Trop,Drain_ drought stress functions were revised. A waterlogging stress function was added to PEATCLSM_Trop,Nat_ to represent reduced transpiration at high water levels in natural tropical peatlands (Hirano et al., 2015). Since artificial drainage consistently lowers the water level to an ideal, vegetation‐dependent level, we did not implement a waterlogging stress function for PEATCLSM_Trop,Drain_.

**Figure 7:**
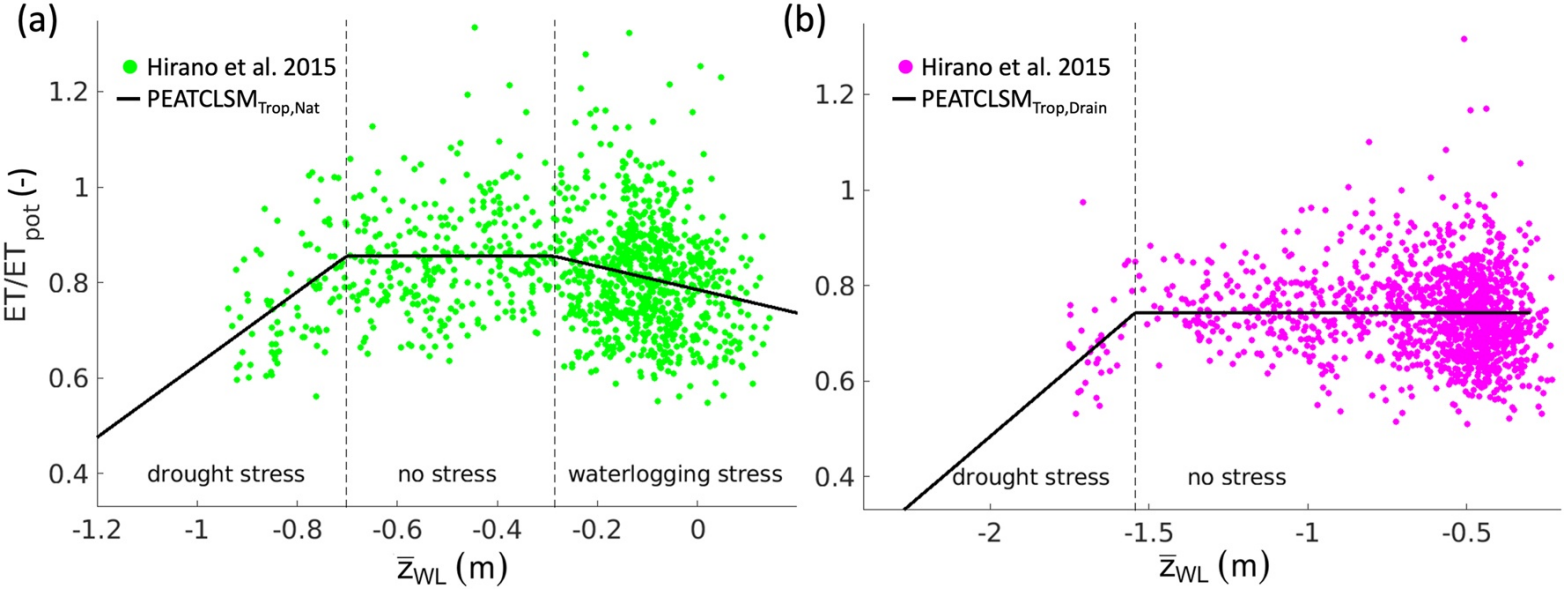
Plant stress functions for both PEATCLSM_Trop_ modules. (a) Derivation of the plant drought and waterlogging stress functions for PEATCLSM_Trop,Nat_ from rescaled daily in situ ET data (ET/ET_pot_; from Hirano et al. (2015) for the period 2004–2007). Plant waterlogging stress occurs at a water level higher than −0.29 m and plant drought stress occurs at water levels lower than −0.70 m. (b) Derivation of the plant drought stress function for PEATCLSM_Trop,Drain_ from ET/ET_pot_ (drained peat swamp forest from Hirano et al. (2015) for the period 2004–2007). Plant drought stress occurs for water levels lower than −1.54 m. ET/ET_pot_ values larger than one are the combined result of ET measurement errors and the imperfect MERRA‐2 derived ET_pot_.

The PEATCLSM_Trop_ plant drought and waterlogging stress functions are shown in Figure 7, and are based on the eddy covariance‐derived ET and water level data (2004–2007) from undrained (Figure 7a) and drained (Figure 7b) peat swamp forests (Hirano et al., 2015), for PEATCLSM_Trop,Nat_ and PEATCLSM_Trop,Drain_, respectively. The net radiation (*R*_net_) data showed a steep, consistent drop during part of the dry season of 2006, probably due to large amounts of haze from peatland fires (Hirano et al., 2015). Therefore, the period covering September 25 through 11 October 2006, was filtered from both ET data sets (drained and undrained peat swamp forest). To limit the seasonal effects of the potential ET (ET_pot_), the in situ ET was rescaled (ET/ET_pot_). The ET_pot_ was calculated with MERRA‐2 data using the method of Priestley and Taylor (1972) as described by Maes et al. (2019). A biome‐specific multiplicative factor (*α*_PT_) of 1.09 (suggested for evergreen broadleaf forests by Maes et al. [2019]) was chosen and is in line with temporal *α*_PT_ values found by Hirano et al. (2015).

For PEATCLSM_Trop,Nat_ (Figure 7a), the plant drought and waterlogging stress function, and the two water level breakpoints were fitted as a piecewise (segmented) linear regression, dividing the data into two stress zones, and one no stress zone. Plant drought stress occurs at water levels lower than −0.70 m, which is turned off with rising water levels and shifts into a plant waterlogging stress function for water levels higher than −0.29 m. For PEATCLSM_Trop,Drain_, the fitted plant drought stress function was obtained through piecewise (segmented) linear regression, with a breakpoint at −1.54 m, dividing the data into a plant drought stress zone at water levels lower than the breakpoint, and a no stress zone for higher water levels (Figure 7b). Despite being the best estimate available, depending on the drained peatland vegetation cover this plant drought stress breakpoint might vary. Comparison of Figures 7a and 7b shows that the mean ET/ET_pot_ in the no stress zone is about 0.1 lower for the drained than the undrained peat swamp forest of Hirano et al. (2015).

In CLSM, the areal fraction for which plant transpiration is shut off (i.e., *F*_wilt_), is defined by the fraction of the spatial root‐zone soil moisture distribution that is at the wilting point. This is not appropriate for peatlands because most water level fluctuations occur in (or close to) the 1‐m root zone of CLSM and a 1‐m root zone is too deep for shallow‐rooted trees in peatlands (Hirano et al., 2015). However, for operational applications of the current CLSM version, making the root‐zone thickness spatially variable would be a too invasive structural change. Therefore, similar to Bechtold et al. (2019), we calculated the *F*_wilt_ using plant drought stress functions that depend on ${\bar{z}}_{WL}$ for PEATCLSM_Trop_. The breakpoints in the PEATCLSM_Trop,Nat_ plant drought stress function (Figure 7a) were used to link *F*_wilt_ and ${\bar{z}}_{WL}$ as follows:

(6) $$\begin{array}{c} \begin{array}{cccc} F_{wilt} & = & 0, & if\;{\bar{z}}_{WL}>-0.70\;m\\ & = & -0.89{\bar{z}}_{WL}-0.63, & if\;-0.70\;m\geq {\bar{z}}_{WL}>-1.82\;m\\ & = & 1, & if\;{\bar{z}}_{WL}\leq -1.82\;m \end{array} \end{array}$$

and for PEATCLSM_Trop,Drain_ the plant drought stress function was implemented as:

(7) $$\begin{array}{c} \begin{array}{cccc} F_{wilt} & = & 0, & if\;{\bar{z}}_{WL}>-1.54\;m\\ & = & -0.76{\bar{z}}_{WL}-1.18, & if\;-1.54\;m\geq {\bar{z}}_{WL}>-2.85\;m\\ & = & 1. & if\;{\bar{z}}_{WL}\leq -2.85\;m \end{array} \end{array}$$

The PEATCLSM_Trop,Nat_ waterlogging stress function was implemented as an additional environmental stress term in the canopy resistance (*r*_*c*_) calculation (Equation 8; Koster & Suarez, 1996). The unstressed canopy resistance (*r*_*c*−unstressed_) is the resistance to plant transpiration in optimal environmental conditions (Koster & Suarez, 1996). The *r*_*c*−unstressed_ is a function of land cover‐type dependent parameters and photosynthetically active radiation. In non‐optimal conditions, environmental stress terms are smaller than one and increase the *r*_*c*_, reducing the vegetation transpiration. Adding the waterlogging stress term resulted in the following equation for the *r*_*c*_ calculation:

(8) $$r_{c}=r_{c-unstressed}F_{temperature}^{-1}F_{waterlogging}^{-1},$$

where *F*_temperature_ is the environmental stress related to temperature, and *F*_waterlogging_ is the waterlogging stress function that was implemented as:

(9) $$\begin{array}{cccc} F_{waterlogging} & = & 1, & if\;{\bar{z}}_{WL}\leq -0.29\;m\\ & = & 1-\frac{\left(0.29+{\bar{z}}_{WL}\right)}{0.64}, & if\;-0.29\;m<{\bar{z}}_{WL}\leq 0.35\;m\\ & = & 0, & if\;{\bar{z}}_{WL}>0.35\;m \end{array}$$

showing that waterlogging stress initiates at a water level of −0.29 m and linearly changes to zero (note the use of *F*_waterlogging_ in the calculation of *r*_*c*_) when the water level reaches 0.35 m.

The slope and range of the waterlogging stress function in Equation 9 and Figure 7a are different, because the waterlogging stress function applied in the *r*_*c*_ calculation (Equation 9) only accounts for a plant transpiration reduction, whereas the waterlogging stress function in Figure 7a shows a plant transpiration reduction that is partially compensated by an increased soil evaporation. The soil evaporation increase only partially compensates the plant transpiration reduction because this evaporation does not occur from a free‐standing water surface but underneath a (dense) canopy layer, and is therefore smaller than the plant transpiration reduction. Because of this difference between the waterlogging stress function in Figure 7a and in Equation 9, the latter was adjusted. The breakpoint at which waterlogging stress initiates (−0.29 m) was kept but the range over which the waterlogging stress occurred was set to 0.64 m, which is four times the microtopographic standard deviation used in PEATCLSM_Trop,Nat_ (0.16 m), because a water level of 0.35 m corresponds to waterlogging of almost all hummocks (Figure 4a).

### 2.3 Study Region and Model Setup

The three study regions of this research cover the major tropical peatland regions in Central and South America, the Congo Basin, and Southeast Asia, shown in Figure 3. For each of the three study regions, simulations with CLSM and PEATCLSM_Trop,Nat_ were conducted. Over Southeast Asia, an additional simulation with PEATCLSM_Trop,Drain_ was performed to account for the large fraction of drained tropical peatlands there. An additional simulation with the PEATCLSM_North,Nat_ model setup from Bechtold et al. (2019) was conducted, but with vegetation input parameters that pertain to the three tropical regions, i.e., including the mean seasonal cycle of satellite‐based LAI (vegetation input parameter) and the broadleaf evergreen land cover type (instead of needleleaf trees and grassland input used in Bechtold et al. [2019]). Table 1 shows an overview of the model configurations, relevant parameters, and boundary conditions for CLSM and the three PEATCLSM modules.

**Table 1:** Overview of the Configurations, Land Model Parameters, and Boundary Conditions for the CLSM, PEATCLSM_North,Nat_, PEATCLSM_Trop,Nat_, and PEATCLSM_Trop,Drain_ Model Versions Used Here

Model version	CLSM	PEATCLSM_North,Nat_	PEATCLSM_Trop,Nat_	PEATCLSM_Trop,Drain_
Soil hydraulic parameters	*θ*_*S*_ = 0.80 m^3^ m^−3^, *h*_S_ = −1.76 m, *b* = 3.41, K_S_ = 7.86 × 10^−7^ m s^−1^	*θ*_*S*_ = 0.93 m^3^ m^−3^, *h*_S_ = −0.03 m, *b* = 3.5, K_S_ = 2.8 × 10^−5^ m s^−1^, K_S,macro,z=0_ = 10 m s^−1^	*θ*_*S*_ = 0.88 m^3^ m^−3^, *h*_S_ = −0.024 m, *b* = 7.4, K_S_ = 6 × 10^−5^ m s^−1^, K_S,macro,z=0_ = 7.3 m s^−1^	*θ*_*S*_ = 0.68 m^3^ m^−3^, *h*_S_ = −0.04 m, *b* = 9.6, K_S_ = 2 × 10^−6^ m s^−1^, K_S,hrz_ = 52 m day^−1^
Topography	Macrotopography from HYDRO1k (USGS)	Standard deviation of the microtopographic distribution		
		*σ* = 0.11 m	*σ* = 0.16 m	*σ* = 0.13 m
Discharge parameters	Discharge based on topographic index	Ivanov function *c* = 1.5 × 10^−5^ m^−1^, *α* = 3 (also uses K_S,macro,z=0_)	Ivanov function *c* = 1.5 × 10^−5^ m^−1^, *α* = 3 (also uses K_S,macro,z=0_)	Dupuit‐Forchheimer function *L*_ditch_ = 0.031 8 m m^−2^, *w*_strip_ = 31.4 m, *z*_ditch_ = −0.68 m
Water‐related stress functions	Drought stress based on root‐zone moisture	Drought stress based on water level	Drought and waterlogging stress based on water level	Drought stress based on water level
Meteorological forcing	MERRA‐2 (Gelaro et al., 2017) including gauge‐based *P* corrections (Reichle, Liu, et al., 2017)			
Land Cover	USGS Global Land Cover Characteristics Data Base Version 2.0 (https://lta.cr.usgs.gov/glcc/)			
Leaf Area Index	Hybrid of Moderate Resolution Imaging Spectroradiometer and GEOLAND2 (Baret et al., 2013; Camacho et al., 2013)			
Greenness fraction	GSWP‐2 (Dirmeyer et al., 2002)			
Peatland distribution map	Hybrid of PEATMAP (Xu et al., 2018) and HWSD1.21 (De Lannoy et al., 2014) distributions			

All simulations were separately spun up for 10 years (from 1 January 1990 through 31 December 1999), which is sufficient to reach equilibrium for tropical peatland regions (data not shown). The subsequent daily output from 1 January 2000 through 31 October 2020 was used for evaluation. All simulations were run at a spatial resolution of 9‐km on the Equal Area Scalable (EASE) grid, version 2.0 (Brodzik et al., 2012). To determine whether a grid cell was peat or not, we used a peatland distribution that is a combination of the PEATMAP distribution from Xu et al. (2018) and peat distribution of De Lannoy et al. (2014) that, over tropical latitudes, corresponds to the Harmonized World Soil Database version 1.21 (HWSD1.21). A 9‐km pixel was entirely treated as peat when the combined peat fraction, for that pixel, was greater or equal to 0.5. Meteorological forcing was taken from the hourly 0.5° × 0.625° (latitude‐by‐longitude) resolution MERRA‐2 reanalysis product with gauge‐based *P* corrections (Reichle, Liu, et al., 2017). Over tropical regions, the MERRA‐2 meteorological forcing data, *P* in particular, are prone to larger errors than in other regions (Reichle, Draper, et al., 2017; Reichle, Liu, et al., 2017), and this will inevitably affect the accuracy of our simulations.

### 2.4 Model Evaluation

#### 2.4.1 In Situ Observations

An extensive data set with in situ observations from all three study regions (Figure 3; and Table B1) was compiled to evaluate water level and ET estimates from the CLSM, PEATCLSM_North,Nat_ and PEATCLSM_Trop_ simulations. The evaluation data sets consist of the following sites in natural peatlands: five sites (1 with eddy covariance data) in Central and South America, four sites in the Congo Basin, and 30 (1 with eddy covariance data) in Southeast Asia. Furthermore, 57 sites (1 with eddy covariance data) were available for drained peatlands in Southeast Asia. The five sites in Central and South America and the four sites in the Congo Basin are the result of averaging water level data from multiple sites within local clusters of highly correlated water level time series. The local averaging ensured that over the data‐sparse regions (Central and South America, and the Congo Basin) the model evaluation is regionally more balanced. The eddy covariance‐derived ET data of the two Southeast Asian sites (the undrained and drained peat swamp forests from Hirano et al. [2015]) was used to derive the plant drought and waterlogging stress functions in Section 2.2.5. It was also used (same period but including the haze period of 2006, see Section 2.2.5) to evaluate model ET improvements for these sites.

The evaluation data set was established from peer‐reviewed literature data, either obtained through direct contact with the authors or manual digitization from the literature source, or from publicly available databases. The “Wild Fire and Carbon Management in Peat‐Forest in Indonesia” project from the Science and Technology Research Partnership for Sustainable Development (SATREPS) provides publicly available, frequently updated water level data (http://kalimantan88.sakura.ne.jp/fire2015/fire2015home.html) that was manually digitized. Real‐time (at daily, hourly, or sub‐hourly temporal resolution) water level data for peatlands in Indonesia are available from the “Sistem Pemantauan Air Lahan Gambut” (SIPALAGA) project (https://sipalaga.brg.go.id/), and were obtained daily since 4 February 2019. The eddy covariance‐derived ET data from the Quistococha palm swamp forest reserve in Peru (73°19′8″W, 3°50′4″S) were obtained from the AmeriFlux network (https://ameriflux.lbl.gov/sites/siteinfo/PE-QFR).

The various external data sources provide data of different quality. Data from peer‐reviewed literature, the SATREPS project, and AmeriFlux were assumed to be quality checked. The water level data from each monitoring site of the SIPALAGA project were manually quality checked, discarding clearly unreliable sites or periods of data. The retained SIPALAGA sites were classified as natural or drained based on Google Earth images, and uncertain sites were left out. If the surface reference height (hollow, hummock, or somewhere in between) of the water level measurements was available, it was, if necessary, shifted to the model surface reference height (mean between hummocks and hollows) using the microtopographic standard deviation for natural and drained peatlands from Section 2.2.2. If no information on the surface reference height of the water level measurements was available, the model surface reference was assumed. The temporal frequency of the water level data ranged from consistent sub‐daily to irregular weekly measurements. Sub‐daily measurements were averaged to daily data and all water level data were compared to daily averaged model output. All eddy covariance‐derived ET data were half‐hourly measurements. The half‐hourly latent heat measurements (W m^−2^) were converted to ET measurements (mm (30 min)^−1^) using a latent heat of water vaporization of 2.43 MJ kg^−1^ and aggregated to daily values. Model evaluation against soil moisture data was not performed due to a lack of sufficient sites with in situ soil moisture time series.

#### 2.4.2 Spatial and Temporal Evaluation

The CLSM and PEATCLSM_Trop_ models were spatially evaluated and compared using 20‐year average (1 January 2000 through 31 December 2019) estimates of hydrological variables for the peat area of all three study regions (Figure 3). Over Southeast Asia, PEATCLSM_Trop,Nat_ and PEATCLSM_Trop,Drain_ were spatially evaluated assuming all peat soil pixels to be natural or drained, respectively. Developing a map that would enable a spatio‐temporal separation of natural and drained peatlands over our 20‐year period was beyond the scope of this paper.

A temporal evaluation was performed for CLSM, PEATCLSM_North,Nat_ and both PEATCLSM_Trop_ versions against in situ observations time series ranged from 2000 to 2020, with different lengths and periods within the time range for various sites. In line with Bechtold et al. (2019), we considered the same five skill metrics:

1. Bias: difference between simulated and observed temporal means (model‐minus‐observation)
2. RMSD: root‐mean‐squared difference between simulated and observed time series
3. ubRMSD: unbiased RMSD, i.e., after removing the bias from the simulated time series
4. R: temporal Pearson correlation coefficient between simulated and observed time series
5. anomR: temporal anomaly Pearson correlation coefficient between simulated and observed data, calculated after removing the mean climatology from the simulated and observed time series. The mean climatology is the multiyear (3‐year minimum) average of 31‐day smoothed time series of daily values. This removal of seasonal correlation due to meteorological forcing allowed us to evaluate the model's interannual and short‐term dynamics.

The requirement of a 3‐year minimum of data to calculate the anomR reduced the number of sites in the water level evaluation to zero in Central and South America, two natural sites in the Congo Basin, and seven natural and four drained sites in Southeast Asia. The anomR was not calculated for ET data. Each skill metric is provided with its 95% CI that takes temporal autocorrelation into account (as in De Lannoy and Reichle [2016]). Skill metrics and CIs were averaged for all sites within a study region, and for Southeast Asia an average of natural and drained sites was calculated separately. The CI averages were divided by the square root of the number of sites per study region, assuming that each site added independent information.

## 3 Results

### 3.1 Spatial Patterns of Hydrological State Variables and Fluxes

#### 3.1.1 Water Level and Soil Moisture

**Figure 8:**
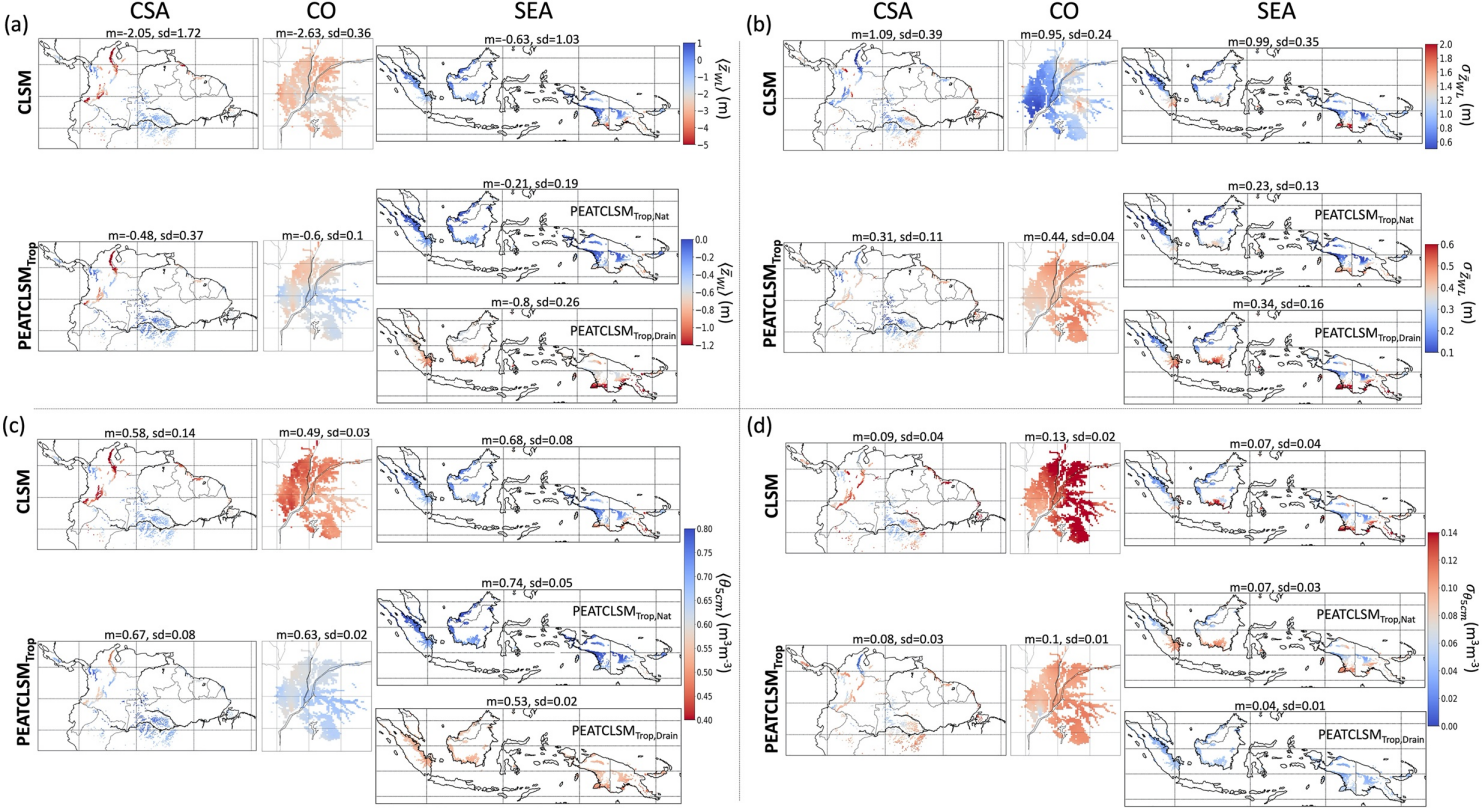
The 20‐year (1 January 2000 through 31 December 2019) (a) mean ${\bar{z}}_{WL}$ $\left(\langle {\bar{z}}_{WL}\rangle \right)$, (b) standard deviation of ${\bar{z}}_{WL}$ $\left(\sigma_{{\bar{z}}_{WL}}\right)$, (c) mean *θ*_5 cm_ (〈*θ*_5 cm_〉), and (d) standard deviation of *θ*_5 cm_ $\left(\sigma_{\theta_{5cm}}\right)$ for CLSM and PEATCLSM_Trop,Nat_ simulations over the three study regions: (left) Central and South America, (middle) the Congo Basin, and (right) Southeast Asia. For Southeast Asia, both PEATCLSM_Trop,Nat_ and PEATCLSM_Trop,Drain_ are shown. The titles show the spatial mean (m) and standard deviation (sd). Note the distinct color bar scales for CLSM and PEATCLSM_Trop_ in (a and b), as well as the inverse color bars in (b and d).

Figure 8 shows the 20‐year mean and standard deviation of ${\bar{z}}_{WL}$ and *θ*_5cm_ for CLSM and PEATCLSM_Trop_ for the peatlands of all three study regions. Figure 8a shows that CLSM simulates lower mean ${\bar{z}}_{WL}$ $\left(\langle {\bar{z}}_{WL}\rangle \right)$ with a larger spatial variation than PEATCLSM_Trop,Nat_ for each region. It also shows that the Congo Basin has the lowest $\langle {\bar{z}}_{WL}\rangle$ and Southeast Asia the highest $\langle {\bar{z}}_{WL}\rangle$ in both simulations. PEATCLSM_Trop,Drain_ simulates a $\langle {\bar{z}}_{WL}\rangle$ of −0.8 m over Southeast Asia. In South America the tropical highland peatlands of the Andes mountains are much drier than surrounding tropical lowland peatlands. Figure 8b illustrates that the temporal standard deviation of ${\bar{z}}_{WL}$ $\left(\sigma_{{\bar{z}}_{WL}}\right)$ over Central and South America decreases from 1.09 m for CLSM to 0.31 m for PEATCLSM_Trop,Nat_. The $\sigma_{{\bar{z}}_{WL}}$ reduction over the Congo Basin is less than over Central and South America, and Southeast Asia, turning the Congo Basin from the region with the lowest $\sigma_{{\bar{z}}_{WL}}$ value (0.95 m) for CLSM to the region with the largest $\sigma_{{\bar{z}}_{WL}}$ value (0.44 m) for PEATCLSM_Trop,Nat_.

The 20‐year mean and standard deviation of *θ*_5 cm_, i.e., 〈*θ*_5cm_〉 and $\sigma_{\theta_{5cm}}$ are shown in Figures 8c and 8d, respectively. The 〈*θ*_5cm_〉 was larger and had smaller spatial variability in PEATCLSM_Trop,Nat_ simulations than in CLSM simulations for every region (Figure 8c), with a 28% increase in 〈*θ*_5cm_〉 over the Congo Basin. For PEATCLSM_Trop,Drain_, the 22% decrease in 〈*θ*_5cm_〉 over Southeast Asia stands out. Figure 8d shows that $\sigma_{\theta_{5cm}}$ slightly decreases over each region from CLSM to PEATCLSM_Trop,Nat_. The $\sigma_{\theta_{5cm}}$ of PEATCLSM_Trop,Drain_ over Southeast Asia is much lower than the $\sigma_{\theta_{5cm}}$ of PEATCLSM_Trop,Nat_ in all three regions.

#### 3.1.2 Runoff Efficiency, Evapotranspiration Efficiency, and Bowen Ratio

**Figure 9:**
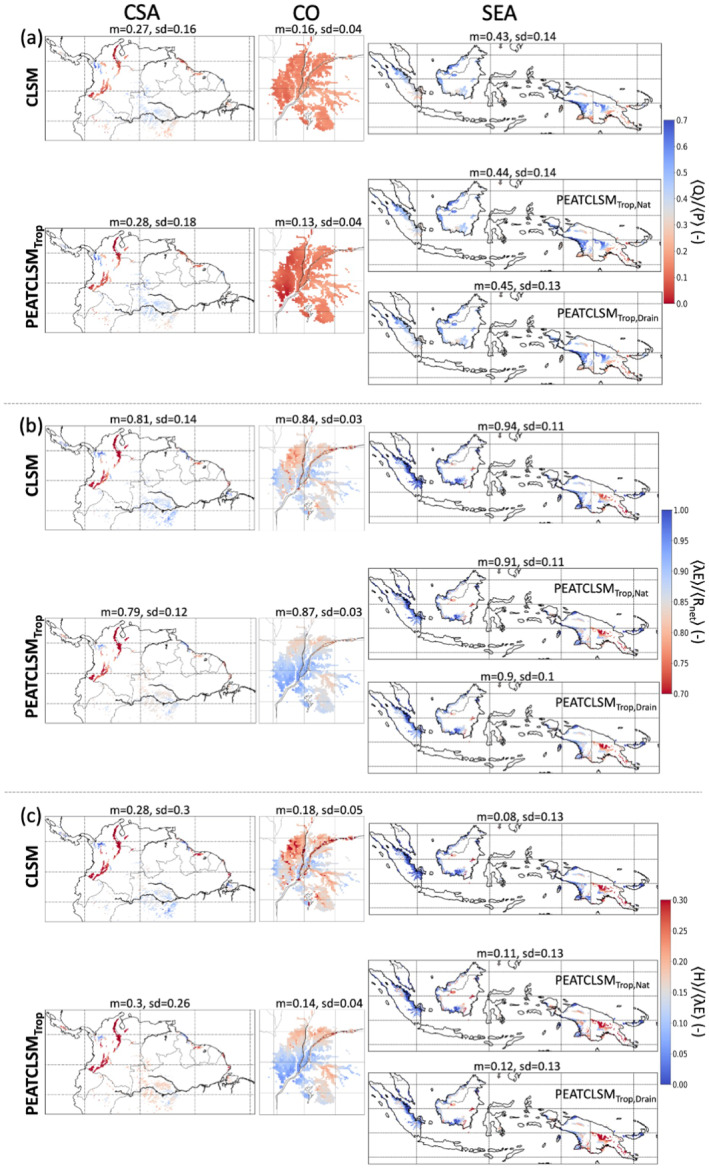
The 20‐year (1 January 2000 through 31 December 2019) mean (a) runoff efficiency (〈*Q*〉/〈*P*〉), (b) evapotranspiration efficiency (〈*λ*E〉/〈*R*_net_〉), and (c) Bowen ratio (〈*H*〉/〈*λ*E〉) for CLSM and PEATCLSM_Trop_ simulations over the three study regions: (left) Central and South America, (middle) the Congo Basin, (right) Southeast Asia. For Southeast Asia, both PEATCLSM_Trop,Nat_ and PEATCLSM_Trop,Drain_ are shown. The titles provide the spatial mean (m) and standard deviation (sd). Note the inverse color bar in (c).

Tropical ombrotrophic lowland peatlands mostly receive water and nutrient input through *P*. Because the change in water storage becomes negligible compared to ET and total runoff (*Q*; both surface and subsurface runoff) over long time scales, the long‐term partitioning of *P* into ET and *Q* determines the water balance, and thus the local hydrologic behavior. The link between long‐term ET and *Q* is essential in LSMs (Koster, 2015; Koster & Mahanama, 2012; Koster & Milly, 1997). Therefore, Figure 9 shows the spatial patterns of 20‐year mean runoff efficiency (〈*Q*〉/〈*P*〉; Figure 9a), evapotranspiration efficiency (〈*λ*E〉/〈*R*_net_〉; Figure 9b), and Bowen ratio (〈H〉/〈*λ*E〉; Figure 9c). Despite substantial changes in ${\bar{z}}_{WL}$, PEATCLSM_Trop_ only marginally changes the three flux ratios over Central and South America, and Southeast Asia. The Congo Basin already had the smallest 〈*Q*〉/〈*P*〉 for CLSM, and the value further decreases by 19% in PEATCLSM_Trop,Nat_ (Figure 9). This decrease is in line with the other ratios for the Congo Basin indicating a smaller *Q* and complementary larger ET.

### 3.2 Evaluation With Field Observations

#### 3.2.1 Water Level

**Figure 10:**
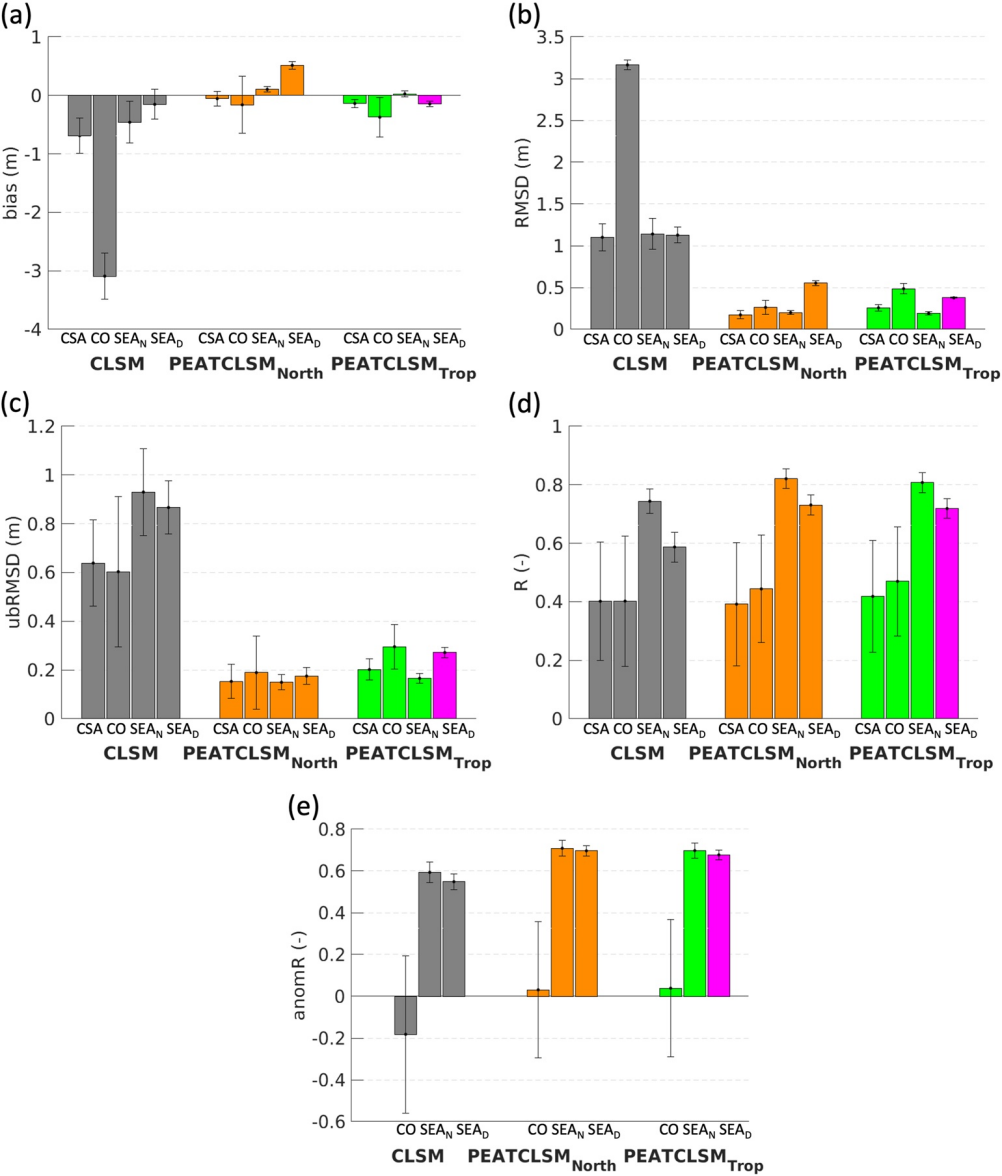
The water level (a) bias, (b) root‐mean‐squared difference (RMSD), (c) unbiased root‐mean‐squared difference (ubRMSD), (d) time series correlation coefficient (*R*), and (e) anomaly time series correlation coefficient (anomR) with the 95% CI for CLSM, PEATCLSM_North,Nat_, and PEATCLSM_Trop_ simulations (PEATCLSM_Trop,Nat_ (green) and PEATCLSM_Trop,Drain_ (pink) over natural and drained sites, respectively), evaluated separately for each study region: Central and South America (CSA), the Congo Basin (CO), and natural (SEA_N_) and drained (SEA_D_) peatlands in Southeast Asia. The evaluation sites and their skill metrics are shown in Tables B1 and B2, respectively.

Figure 10 presents the average model skill metrics at evaluation sites with water level data (Figure 3; Table B1). Data from 39 sites in natural peatlands are used to evaluate CLSM, PEATCLSM_North,Nat_, and PEATCLSM_Trop,Nat_, whereas data from 57 sites in drained peatlands are used to evaluate CLSM, PEATCLSM_North,Nat_, and PEATCLSM_Trop,Drain_. The skill metrics for the CLSM and PEATCLSM_Trop_ simulations for each of the 96 sites with water level data are provided in Table B2.

A large bias, RMSD and ubRMSD for CLSM (Figure 10) confirm that CLSM simulates an average ${\bar{z}}_{WL}$ that is too low in Central and South America, and the Congo Basin, and fluctuations in ${\bar{z}}_{WL}$ that are too large in all three regions. PEATCLSM_Trop_, as well as PEATCLSM_North,Nat_, drastically reduces the average bias, ubRMSD and RMSD, and their corresponding CIs for all regions. CLSM has an extremely large average bias and RMSD over the Congo Basin that is strongly improved by PEATCLSM_Trop_, but the model skill of PEATCLSM_Trop,Nat_ for the Congo Basin remains considerably worse than for the other regions. PEATCLSM_North,Nat_ slightly outperforms PEATCLSM_Trop,Nat_ over the Congo Basin with a smaller absolute bias, RMSD, and ubRMSD. However, over Southeast Asia, the absolute bias was smaller compared to PEATCLSM_North,Nat_. PEATCLSM_North,Nat_ and PEATCLSM_Trop,Drain_ had similarly improved the simulations over CLSM for the drained sites in Southeast Asia, but PEATCLSM_Trop,Drain_ did additionally reduce the absolute bias by 0.37 m compared to PEATCLSM_North,Nat_. In terms of *R*, PEATCLSM_Trop_ improves the skill compared to CLSM over Central and South America, the Congo Basin, natural sites in Southeast Asia, and drained sites in Southeast Asia, resulting in a *R* improvement of 0.02, 0.07, 0.07, and 0.13, respectively (Figure 10d). Figure 10e shows that PEATCLSM_Trop_ significantly improves the anomR for natural (0.73) and drained (0.68) sites in Southeast Asia, though the average anomR over the Congo Basin remained low (0.04), which is likely due to the poor meteorological forcings over this region.

**Figure 11:**
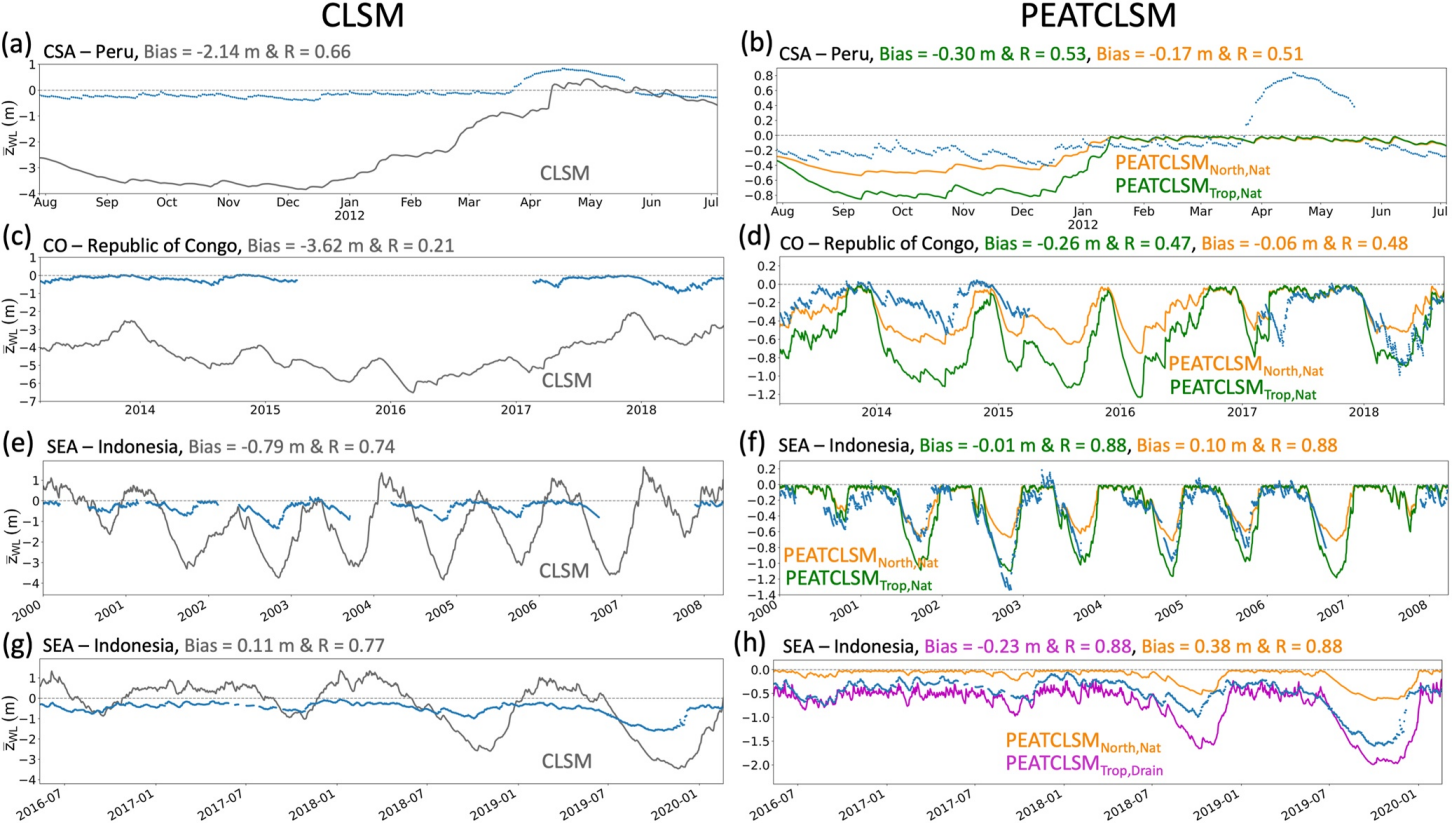
Comparison of in situ water level (blue dots) to (a, c, e, and g) CLSM and (b, d, f, and h) PEATCLSM simulated ${\bar{z}}_{WL}$ for: (a and b) a site in Peru (73°19′8″W, 3°50′24″S), (c and d) a site in the Republic of the Congo (17°28′42″E, 1°12′46″N), (e and f) a natural site in Indonesia (114°6′0″E, 2°25′12″S), and (g and h) a drained site in Indonesia (114°3′29″E, 2°19′12″S). CLSM simulations are gray, PEATCLSM_North,Nat_ simulations are orange, PEATCLSM_Trop,Nat_ simulations are green, and PEATCLSM_Trop,Drain_ simulations are purple.

To illustrate model and regional differences in simulated ${\bar{z}}_{WL}$ dynamics, a comparison against water level timeseries from a representative evaluation site for each region (for Southeast Asia both a natural and drained site) is shown in Figure 11. The sites had to span at least 1 year of data and be in line with the average model skill metrics for that region. Once again, the unrealistic ${\bar{z}}_{WL}$ fluctuations (both positive and negative) of CLSM stand out for each site. Figures 11e and 11g show that CLSM simulates long periods of ${\bar{z}}_{WL}>$ 0 m. In CLSM, values of ${\bar{z}}_{WL}>$ 0 m do not represent real flooding as CLSM does not allow water to pond at the surface, but instead it indicates that a large fraction of the soil in the pixel is saturated. In situ data shows flooding only for the site in Figure 11a. By contrast, PEATCLSM_Trop_ does not simulate ${\bar{z}}_{WL}>$ 0 m, but only ponding in hollows up to the mean surface elevation (${\bar{z}}_{WL}$ = 0 m). PEATCLSM_Trop_ still simulates too low ${\bar{z}}_{WL}$ during the dry season (timing differs across regions), especially PEATCLSM_Trop,Nat_ over Central and South America, and the Congo Basin, and PEATCLSM_Trop,Drain_ over Southeast Asia. PEATCLSM_North,Nat_ reduces these too deep ${\bar{z}}_{WL}$ during the dry season over Central and South America, and the Congo Basin but simulates too shallow ${\bar{z}}_{WL}$ during the dry season for a natural site in Southeast Asia. Figure 11h shows that PEATCLSM_North,Nat_ consistently overestimates ${\bar{z}}_{WL}$, and is outperformed by PEATCLSM_Trop,Drain_.

#### 3.2.2 Daytime Evapotranspiration

**Figure 12:**
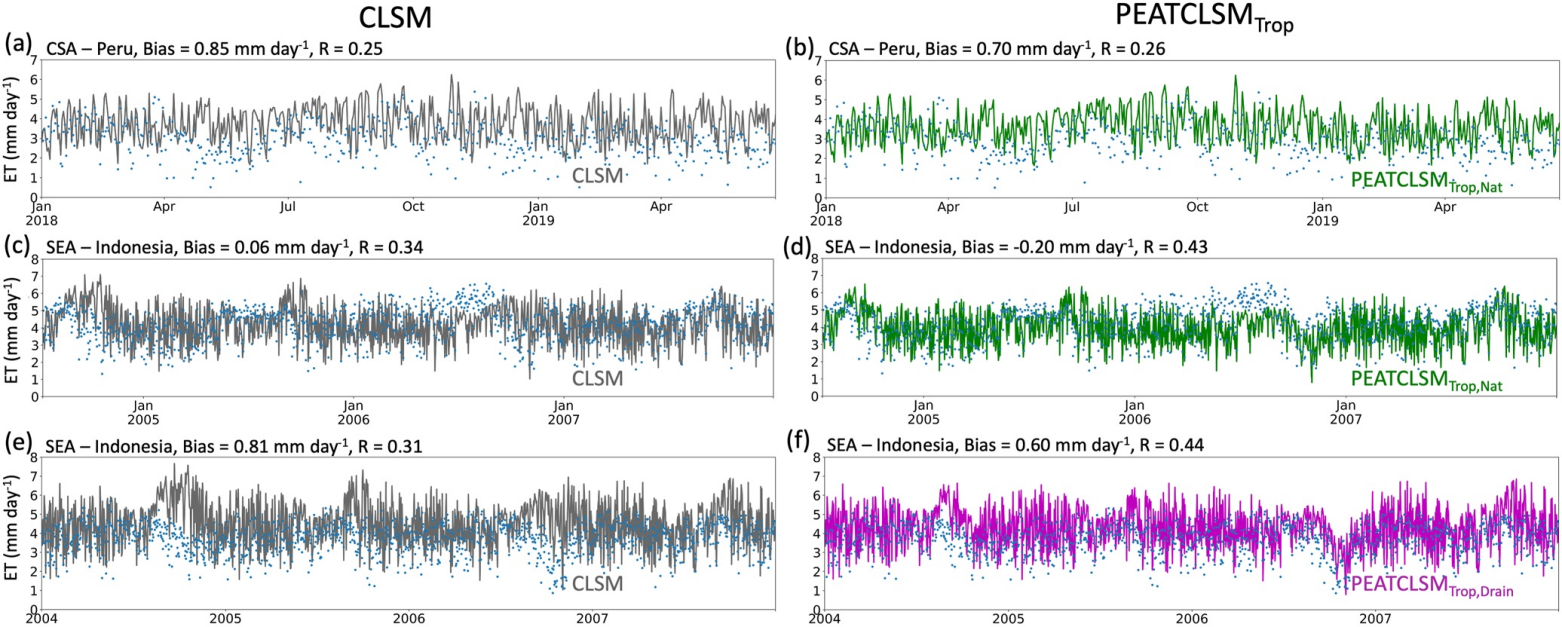
Comparison of in situ ET (blue dots) to (a, c, and e) CLSM and (b, d, and f) PEATCLSM_Trop_ simulated ET for: (a and b) the Quistococha palm swamp forest reserve in Peru (73°19′8″W, 3°50′4″S), (c and d) the undrained peat swamp forest from Hirano et al. (2015) in Indonesia (113°54′29″E, 2°19′19″S), and (e and f) the drained peat swamp forest from Hirano et al. (2015) in Indonesia (114°2′10″E, 2°20′46″S). CLSM simulations are gray, PEATCLSM_Trop,Nat_ simulations are green, and PEATCLSM_Trop,Drain_ simulations are purple.

Only three sites with eddy covariance measurements over tropical peatlands were available to evaluate the ET simulation skill of CLSM, PEATCLSM_North,Nat_ and PEATCLSM_Trop_. Figure 12 compares the daily modeled and observed ET time series for one site in Peru, and a natural and drained site in Indonesia. The ET data of the two sites in Indonesia were also used to derive the PEATCLSM_Trop_ plant stress functions (Section 2.2.5), which should be considered when evaluating model results. For all three sites, PEATCLSM_Trop_ increases the correlation coefficient compared to CLSM, especially at the natural (Figure 12d) and the drained (Figure 12f) sites in Indonesia. PEATCLSM_Trop,Nat_ slightly improved the correlation coefficient for both natural sites compared to PEATCLSM_North,Nat_ (not shown), whereas for the drained site PEATCLSM_Trop,Drain_ and PEATCLSM_North,Nat_ performed equally well. Both CLSM and PEATCLSM_Trop_ simulate too large ET, except for the natural site in Indonesia, where CLSM has a small positive bias of 0.06 mm day^−1^ (Figure 12c), and PEATCLSM_Trop,Nat_ underestimates ET by 0.22 mm day^−1^ (Figure 12d). For the natural and drained site in Indonesia, PEATCLSM_Trop,Nat_ and PEATCLSM_Trop,Drain_ show major improvements in the late dry season of dry (El Niño) years, better following the steep drop of in situ observed ET for the natural and drained site in Indonesia, respectively. PEATCLSM_Trop,Nat_ improves the absolute bias in ET over PEATCLSM_North,Nat_ from 0.82 to 0.70 mm day^−1^ and from −0.24 to −0.22 mm day^−1^ for the natural peatland sites in Peru and Indonesia, respectively. PEATCLSM_North,Nat_ did reduce the absolute bias over PEATCLSM_Trop,Drain_ from 0.51 mm day^−1^ to 0.60 mm day^−1^ for the drained site.

## 4 Discussion

### 4.1 Regional Differences in Model Performance

The Congo Basin appears as the driest simulated region with the largest $\sigma_{{\bar{z}}_{WL}}$ for both CLSM and PEATCLSM_Trop,Nat_ (Figure 8), and with the largest negative water level bias (too dry simulations) compared to in situ data (Figure 10). The area is relatively drier, because the mean annual *P* in the Congo Basin is ±1,700 mm yr^−1^ (Samba & Nganga, 2012), which is considerably lower than other tropical peatland regions (Iquitos, Peru, ±3,000 mm yr^−1^ [Marengo, 1998]; Central Kalimantan, Indonesia, ±2,900 mm yr^−1^ [Susilo et al., 2013]). Furthermore, Figure 11d illustrates that the main dry bias in water level by PEATCLSM_Trop,Nat_ occurs during the dry season. This possibly excludes that the simulations would be too dry due to missed lateral water input from river flooding. Dargie et al. (2017) also indicates that the Congo Basin is mostly fed by P, whereas flooding by rivers is only of secondary importance. Davenport et al. (2020) support the presumption of shallowly domed peatlands in the Congo Basin, making it even more likely to mainly be a rainfed peatland complex. They assume a doming gradient of ±3 m per 40 km, which is a very gentle slope compared to gradients of 20 m per 40 km (Page et al., 1999) or 7 m per 14 km (Cobb et al., 2017) in Southeast Asian peatlands. Assuming similar microtopography and peat properties, a gentler sloped peat dome reduces water flow compared to a peat dome with a steeper gradient, which means that a natural Congolese peat dome has much smaller discharge at high water levels than the PEATCLSM_Trop,Nat_ discharge function derived from an Indonesian peat dome. A separate discharge function could be obtained from new field research or by tuning the current PEATCLSM_Trop,Nat_ discharge function to the water level data. The very low simulated 〈*Q*〉/〈*P*〉 for the Congo Basin (Figure 9a) illustrates that compared to Southeast Asia or Central and South America (apart from the peatlands in the Andes mountain range) the relative simulated *Q* in the Congo Basin is even smaller than expected from the lower *P*. Burnett et al. (2020) estimated the 〈*Q*〉/〈*P*〉 based on a water balance model and obtained a slightly higher average (from 2003 through 2015) value of 0.22 for the entire Congo Basin (including peatlands). Accurate representation of the regional peatland hydrology over the Congo Basin is necessary, especially because the Congolese rainforest is, on average, much drier than the tropical rainforests in Central and South America, and Southeast Asia, making it more water‐limited during the dry season and even more vulnerable to changes in rainfall patterns (Jiang et al., 2019). Besides improved parameterization, more accurate simulations in the Congo Basin will also require an improvement in the meteorological forcing data for this region (see Section 4.4).

The Central and South American peatlands display a lot of variability in the simulated wetness (Figures 8a and 8c), with wet peatlands around the Amazon River and in Central America, but drier peatlands in the northern Andes of Venezuela and Colombia, and at the coastlines of the Guianan moist forest. The tropical highland peatlands in the northern Andes mountains have a very different, and altitude‐dependent, climate, vegetation, and hydrology (Benfield et al., 2021; Chimner et al., 2019) compared to the ombrotrophic lowland peatlands that were used to derive PEATCLSM_Trop,Nat_ parameters. The Andean peatlands have a much lower *P* and a near‐zero *Q*, resulting in the extremely low 〈*Q*〉/〈*P*〉 in Figure 9a. The unrealistically low ${\bar{z}}_{WL}$ and *θ*_5cm_, and the mere fact that PEATCLSM_Trop_ was developed to simulate the hydrology of tropical ombrotrophic lowland peatlands, indicate that this module is not optimal to simulate the diverse hydrology of tropical highland peatlands. However, PEATCLSM_Trop,Nat_ did simulate a high average ${\bar{z}}_{WL}$ that is close to the −0.2 m average measured by Benavides (2014) in 13 natural highland tropical peatlands at the Iguaque massif. The in situ water level of the Peruvian site shown in Figures 11a and 11b rises almost 1 m above the surface during the wet season. The discharge function of PEATCLSM_Trop,Nat_ (Figure 6a) limits the ${\bar{z}}_{WL}$ to rise above the mean surface elevation. But for some peatlands, intense rainfall events and river flooding can cause water levels above the mean surface elevation (Lawson et al., 2014). Removal of the flood period for two evaluation sites improved the overall PEATCLSM_Trop,Nat_ skill over Central and South America, increasing *R* from 0.42 to 0.50 and reduced the bias from −0.14 to −0.09 m. Lawson et al. (2014) and Kelly et al. (2014) did mention that flooding of such an extent is exceptional, and that these peatlands might flood up to 0.2 m above the surface during a normal wet season. Only two out of the 29 Southeast Asian evaluation sites over natural tropical peatlands showed temporary surface inundation events that reached heights of about 0.5 m, always at the end of the wet season. Lähteenoja et al. (2009) and Schulz et al. (2019) showed that peatlands in the Peruvian Amazon have a distinct and variable hydrology: some are almost purely rainfed (what we simulate with PEATCLSM_Trop_), others are seasonally flooded for several months or occasionally flooded but mainly rainfed, which is not captured by our global model scheme. Although combining PEATCLSM with information on the surrounding landscape (e.g., river routing as done by Getirana et al. [2012]) could partially overcome the difficulty of parametrizing the influence of external water input in minerotrophic peatlands, the diversity of Amazonian peatlands makes a spatial map that distinguishes between peatland types unlikely to be developed in the near future.

PEATCLSM_Trop,Drain_ decreased $\langle {\bar{z}}_{WL}\rangle$ and 〈*θ*_5cm_〉 compared to CLSM in Southeast Asia, whereas the PEATCLSM_Trop,Nat_ increased the wetness in all regions. Both improvements better correspond with water level data from evaluation sites. The decrease in $\langle {\bar{z}}_{WL}\rangle$ for PEATCLSM_Trop,Drain_ is partly due to a dry‐season overestimation of *R*_net_ (see Section 4.4). A reduction in *θ*_5cm_ for PEATCLSM_Trop,Drain_ was also expected from the hydraulic properties and discharge function (Figure 6b), preventing the ${\bar{z}}_{WL}$ from reaching values much higher than −0.4 m (Table 1). This −0.4 m “limit” results in much smaller *θ*_5cm_ fluctuations, which translates into a $\sigma_{\theta_{5cm}}$ value for PEATCLSM_Trop,Drain_ that is much lower than all other $\sigma_{\theta_{5cm}}$ values. Hooijer et al. (2012) showed that peat drainage increases bulk density (i.e., decreases porosity) up to a depth of ±0.5 m below the surface, but does not have a strong impact on the bulk density of deeper peat layers (shown in Figure 1c).

### 4.2 Model Structure and Parameter Limitations

The regional differences in model performance highlight that a better spatial differentiation between ombrotrophic and minerotrophic peatlands, highland and lowland peatlands, and the inclusion of lateral water input from river flooding could improve the simulations. The well‐studied peatlands in Southeast Asia are mostly ombrotrophic domes (Page et al., 2006), but a great diversity of tropical peatland types in the less well‐studied regions of Central and South America and Africa is likely (Dargie et al., 2017; Lähteenoja et al., 2009).

Although the degree of artificial drainage varies spatially and in time, we approximated the effects of drainage using a single set of representative parameters, similar to how vegetation with different surface energy exchange characteristics is combined in a single LSM land cover type. The discharge function of PEATCLSM_Trop,Drain_ (see Section 2.2.4, and Figure 6b) was developed using information on drainage canals in Southeast Asian peatlands (Dadap et al., 2021). This map of drainage canals could be used to develop a spatially varying discharge function for PEATCLSM_Trop,Drain_, but also to spatially distinguish between natural and drained peatlands using a threshold. However, the map only represents current drainage canals and doesn't take local canal management into account. Although land use has been mapped over time (Miettinen et al., 2016), drainage is not always well‐coordinated with it (Dadap et al., 2021), making the drainage map's usefulness for long simulation periods uncertain.

In addition to a better horizontal description of land surface processes, a more detailed vertical representation of the peat profile could improve local simulations. A proper description of the peat hydraulic properties in the acrotelm suffices, if water level fluctuations are mainly limited to the top meter (like in natural northern peatlands), but when water level fluctuations in deeper layers occur frequently, deep layer peat properties are needed to accurately describe the hydrological behavior. In natural tropical peatlands, most water level fluctuations occur in the upper 0.5 m of soil, but field data show that during dry seasons the water level can decline to −1.5 m (Figure 11f). Similar and even larger fluctuations occur in drained peatlands and here the large differences in peat properties between upper and lower peat layers result in a different hydrology. Including depth‐specific soil properties in PEATCLSM_Trop_ could partially reduce the too low simulated ${\bar{z}}_{WL}$ during the dry season (Figures 11b, 11d, 11f, and 11h), and possibly improve the simulation dynamics (e.g., better timing of ${\bar{z}}_{WL}$ rise during dry season) even further. However, even if such a layering were included, our parameter sets consist of “average” parameters derived from a handful of literature sources. Currently, data on peatland properties around the world are insufficient to develop vertically and horizontally differentiated parameter maps, similar to those used for mineral soils.

### 4.3 The Need for a Tropical Peatland‐Specific Model Structure and Parametrization

The additional simulation with PEATCLSM_North,Nat_ allowed an evaluation of the possible benefit of PEATCLSM_Trop_ over PEATCLSM_North,Nat_ for both natural and drained tropical peatlands. PEATCLSM_North,Nat_ and PEATCLSM_Trop,Nat_ similarly improve the skill over CLSM for natural tropical peatlands in all three regions and show similar differences in performance across regions (Figure 10). The differences in ubRMSD, *R* and anomR between PEATCLSM_North,Nat_ and PEATCLSM_Trop_ were minor (Figure 10) because the same basic model structure, meteorological input, and the adoption of the same vegetation input parameters from tropical peatlands were applied in the PEATCLSM_North,Nat_ simulations. The newly implemented structural changes (i.e., waterlogging stress in PEATCLSM_Trop,Nat_, and the Dupuit‐Forchheimer discharge function in PEATCLSM_Trop,Drain_) and parameter updates of PEATCLSM_Trop_ did not induce major improvements in the water level skill metrics compared to PEATCLSM_North,Nat_.

Despite the fact that the overall improvements of PEATCLSM_Trop_ over PEATCLSM_North,Nat_ are minor, it can be argued that PEATCLSM_Trop_ is more appropriate and has a more robust structure in certain circumstances and for specific output variables. PEATCLSM_Trop_ reduced absolute water level bias compared to PEATCLSM_North,Nat_ over both natural and drained tropical peatlands in Southeast Asia (Figure 10). This reduction occurs in particular during dry periods (Figures 11f and 11h), when peatlands are most vulnerable and accurate water level simulations are crucial for fire risk and carbon modeling. Except for the bias of the drained site, PEATCLSM_Trop_ outperformed PEATCLSM_North,Nat_ in the ET evaluation (Section 3.2.2). The main improvements of PEATCLSM_Trop,Nat_ over PEATCLSM_North,Nat_ occurred at the beginning of the dry season due to the adapted *F*_wilt_ (Section 2.2.5); however, more eddy covariance data is needed to properly evaluate this. The simulated surface (and, to a lesser extent, root‐zone) soil moisture dynamics differed between PEATCLSM_North,Nat_ and PEATCLSM_Trop_ (not shown) and are likely due to the different hydraulic properties (Figures 5e and 5f). Due to the lack of sufficient in situ measurements, an evaluation of surface or root‐zone soil moisture content was not conducted.

Furthermore, our results show that both PEATCLSM_North, Nat_ and PEATCLSM_Trop,Nat_ perform poorly over Central and South America, and the Congo Basin, whereas the availability of data to parametrize PEATCLSM_Trop_ in Southeast Asia led to a better model performance in this area. This suggests that peatland modules of Earth system models would ideally be specifically developed or tuned for each tropical peatland type or region—and that improvements of PEATCLSM_Trop,Nat_ over PEATCLSM_North,Nat_ in tropical regions outside of Southeast Asia would indeed be seen if adequate data for this regional tuning were available and the necessary structural model changes were made.

### 4.4 Meteorological Forcing Data Uncertainties

Some shortcomings of our simulations are not due to model structure limitations or lack of literature data to constrain parameters, but due to inaccurate meteorological forcing data. The MERRA‐2 gauge‐based corrected *P* is of poor quality over tropical regions, especially over the Congo Basin (Reichle, Draper, et al., 2017; Reichle, Liu, et al., 2017). The low NOAA Climate Prediction Center (CPC) Unified Gauge‐Based Analysis of Global Daily Precipitation (CPCU) gauge count over Africa, resulted in a MERRA‐2 *P* correction with the coarse spatial scale CPC Merged Analysis of Precipitation (CMAP) product for the continent (Bosilovich et al., 2016; Reichle, Liu, et al., 2017). Reichle, Liu, et al. (2017) showed that the mean annual MERRA‐2 observation corrected *P* followed the CPCU gauge count, i.e., low annual *P* in years with low CPCU gauge count, and vice versa. Despite the rather constant gauge count over time, the very low gauge density resulted in an average spacing of 400 km between gauges in Central Africa, which is far from sufficient in a region dominated by convective (high spatial variation) rainfall (Reichle, Liu, et al., 2017). Comparison of PEATCLSM_Trop,Nat_ ${\bar{z}}_{WL}$ time series against in situ water level revealed that sometimes the simulated ${\bar{z}}_{WL}$ reaches the surface at the start of the wet season with a delay of about a month. This occurred when dry season simulated ${\bar{z}}_{WL}$ was too low, but also when the dry season simulated ${\bar{z}}_{WL}$ was reasonably accurate or even too high. The initiation and drawdown of the simulated ${\bar{z}}_{WL}$ is in line with, and at a similar pace as, that of the in situ water level data, and so is the initiation of the simulated ${\bar{z}}_{WL}$ rise. However, when large, local *P* events at the beginning of the water level rise are not well captured by the coarse resolution of MERRA‐2, the pace of the simulated ${\bar{z}}_{WL}$ rise becomes too slow. An evaluation of uncertainties in PEATCLSM_Trop_ model predictions caused by uncertainty in forcing data is left for future research.

Inaccurate meteorological variables that drive ET, resulted in additional uncertainties for the PEATCLSM_Trop,Drain_ simulation. Figure 11h displayed an underestimation by PEATCLSM_Trop,Drain_ simulated ${\bar{z}}_{WL}$ during the dry season, for one specific site. However, this PEATCLSM_Trop,Drain_ dry season underestimation occurs for most sites, and strongly contributes to the average negative bias of −0.15 m over 57 evaluation sites (Figure 10a) for PEATCLSM_Trop,Drain_. Comparison of PEATCLSM_Trop,Drain_ simulated ET to eddy covariance‐derived ET (Figure 12f) showed a slight model overestimation during the wet season, and despite the improvements compared to CLSM, PEATCLSM_Trop,Drain_ strongly overestimated ET during the dry season. For the drained peat swamp forest site from Hirano et al. (2015) the model (MERRA‐2) *R*_net_ and vapor pressure deficit are on average (2004 through 2007) 7.79 W m^−2^ (5.2%) and 0.22 kPa (28.2%) lower than the in situ data, which should indicate lower model than eddy covariance‐derived potential ET and does not explain the underestimation of ${\bar{z}}_{WL}$.

**Figure 13:**
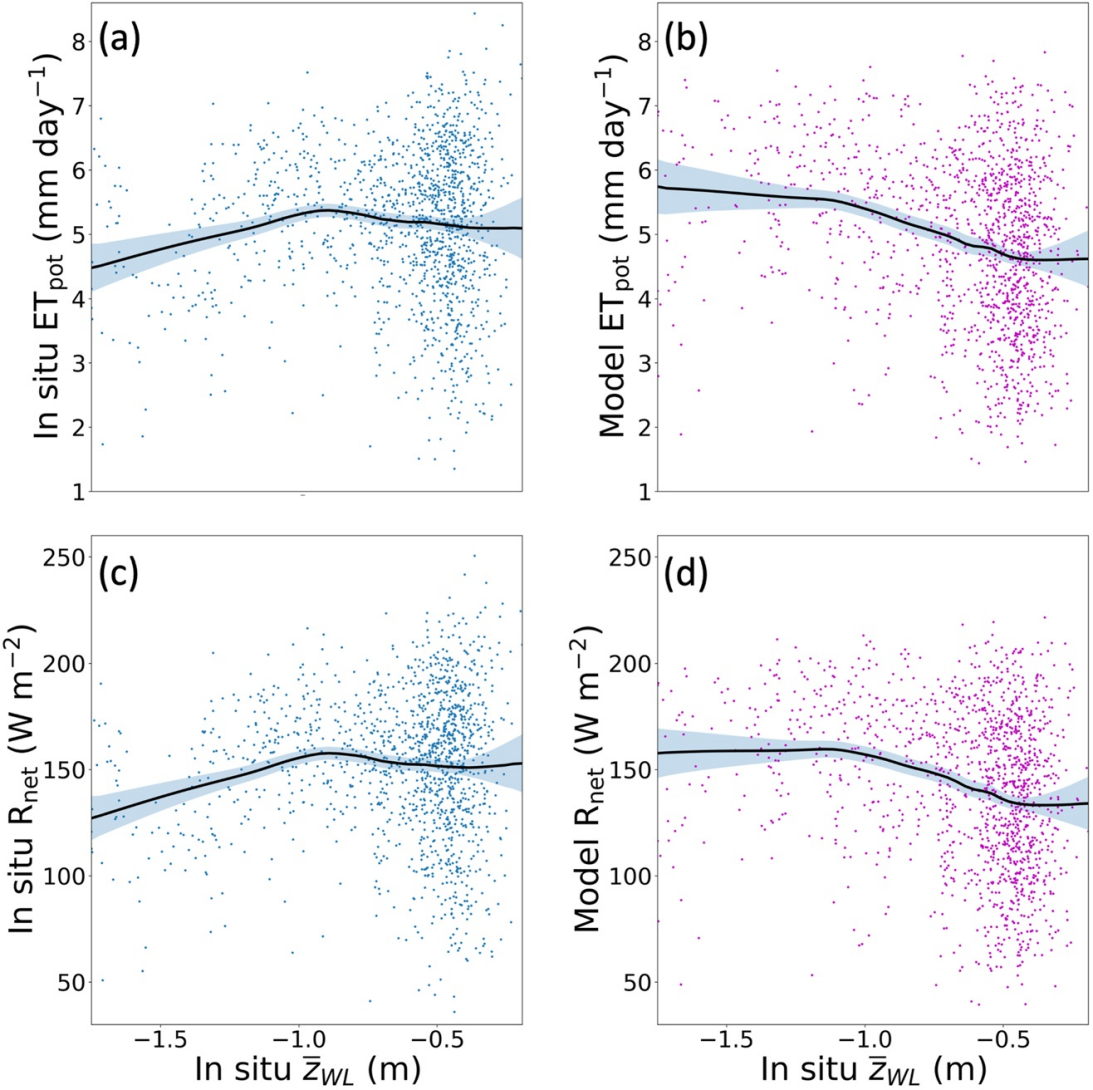
Comparison of the (a) in situ and (b) model ET_pot_, and (c) in situ and (d) model net radiation (*R*_net_) to the in situ water level (m) for the drained peat swamp forest from Hirano et al. (2015) (114°2′10”E, 2°20′46”S). Daily values for four years (from 1 January 2004 through 31 December 2007) are shown together with the locally weighted scatterplot smoothing (LOWESS) fit (black line) and corresponding 95% CI (blue area).

Further analysis of the meteorological variables that drive ET provided insight into this discrepancy. Figure 13 compares the in situ and model ET_pot_, and in situ and model *R*_net_ against the in situ measured water level for the drained peat swamp forest from Hirano et al. (2015) for the period 2004 through 2007. We used the Priestley‐Taylor method to estimate ET_pot_ based on in situ and simulated temperature, as explained in Section 2.2.5. A locally weighted scatterplot smoothing (LOWESS) fit and corresponding 95% CI (using bootstrapping) were calculated for each subplot of Figure 13. The model *R*_net_ and ET_pot_ in the wet season (high water level) are slightly underestimated, but the strong decrease in observed *R*_net_ and ET_pot_ in the dry season (low water level) is not captured by the model forcing data, which reaches its highest *R*_net_ and ET_pot_ values in the late dry season. Hirano et al. (2015) concluded that the in situ observed *R*_net_ (and resulting ET_pot_) decrease was due to smoke or haze. When comparing the haze‐induced reduction of *R*_net_ with MERRA‐2, we can see that this reduction is not captured.

Aerosol emissions from biomass burning in MERRA‐2 are derived from the Reanalysis of the Tropospheric Chemical Composition, version 2 (Schultz et al., 2008), the Global Fire Emissions Database, version 3.1 (van der Werf et al., 2006), and the Quick Fire Emission Data set, version 2.4r6 (QFED‐2.4.r6; Darmenov & da Silva, 2015). According to Darmenov and da Silva (2015), emissions from smoldering and peat fires with low thermal signature are not well captured, resulting in an underestimation of the QFED‐2.4.r6 over Southeast Asia. They refer to the large‐scale fires in the dry season of 2006 (also see Figures 11f and 12), and the difficulty that QFED‐2.4.r6 has with capturing the extent of such an extreme event in peatlands. This underestimation of aerosols in MERRA‐2 for smoldering peat fires results in an overestimation of ET_pot_ and thus adds to the ${\bar{z}}_{WL}$ dry‐bias during the dry season.

PEATCLSM_Trop_ improves the ET simulations for the three eddy covariance sites. An increase in *R* and a decrease in the high positive bias, except for a slightly larger negative bias in Figure 12d, clearly illustrates that for these three sites PEATCLSM_Trop_ outperforms CLSM. However, no robust conclusions about ET dynamics can be drawn based on only three evaluation sites, that cover a limited time range, and given the fact that the data from the two sites over Southeast Asia were also used to derive the plant drought and waterlogging stress functions (Section 2.2.5).

## 5 Conclusions

The original PEATCLSM module (i.e., PEATCLSM_North,Nat_) was developed by Bechtold et al. (2019) to include the peat‐specific land surface hydrology of ombrotrophic natural northern peatlands in the GEOS CLSM. In this research, we adapted and extended the PEATCLSM_North,Nat_ module to better simulate the hydrology of natural (PEATCLSM_Trop,Nat_) and drained (PEATCLSM_Trop,Drain_) tropical peatlands. Literature‐based parameter sets for both PEATCLSM_Trop_ modules were developed without parameter tuning, and two structural changes were realized. The PEATCLSM_Trop,Nat_ module was extended with a plant waterlogging stress function to describe reduced plant transpiration at very high water levels, and the PEATCLSM_Trop,Drain_ discharge was described using the Dupuit‐Forchheimer function. PEATCLSM_Trop_ is the first large‐scale hydrological LSM scheme for tropical peatlands.

The development of model parameters and robust evaluation for tropical peatlands is restricted by the limited data availability. Nevertheless, PEATCLSM_Trop_ parameter sets were developed with data from tropical ombrotrophic lowland peatlands in Southeast Asia, and an evaluation data set of water level and ET measurements in Central and South America, the Congo Basin and Southeast Asia was compiled. Recent global peatland mapping efforts (Gumbricht et al., 2017; Xu et al., 2018), the description of the Cuvette Centrale peatland complex in the Congo Basin (Dargie et al., 2017), and the recognition of the value and mitigation potential of tropical peatlands (Leifeld & Menichetti, 2018; Loisel et al., 2021; Page, Rieley, & Banks, 2011; Wijedasa et al., 2017) might accelerate much‐needed research and data collection over tropical peatlands, especially in Central and South America, and the Congo Basin, in the near future.

PEATCLSM_Trop,Nat_, PEATCLSM_North,Nat_ and CLSM simulations were run from 2000 through 2020 over three study regions, i.e., for peatlands in Central and South America, the Congo Basin and Southeast Asia, and supplemented with a PEATCLSM_Trop,Drain_ simulation over Southeast Asia. A comparison of 20‐year averaged spatial patterns of hydrological variables, and an evaluation against in situ water level and ET data over all three study regions showed that:

1. CLSM simulated too low ${\bar{z}}_{WL}$ with unrealistic fluctuations, which were strongly reduced in PEATCLSM_Trop_ simulations (Figures 8a and 8b);
2. PEATCLSM_Trop_ skill strongly differed between regions, although improvements relative to CLSM were generally comparable for all regions;
3. Both CLSM and PEATCLSM_Trop,Nat_ simulated the lowest ${\bar{z}}_{WL}$ and *θ*_5cm_ for the Congo Basin;
4. The large variability of simulated hydrological variables within Central and South American peatlands mainly relate to spatial climate variability for the different regions; and
5. PEATCLSM_Trop,Drain_ improved dynamics of both ${\bar{z}}_{WL}$ and *θ*_5cm_ simulations, which results in a lower water level ubRMSD and RMSD, and higher *R* at drained sites than for CLSM. The bias is also strongly reduced compared to PEATCLSM_North,Nat_ and PEATCLSM_Trop,Nat_.

All PEATCLSM_Trop_ parameter sets were derived from data collected in Southeast Asian ombrotrophic lowland peatlands and may not be representative for all tropical peatland regions. Some parameters might benefit from further global or local tuning as more data becomes available. A full sensitivity analysis is left for future research. Furthermore, rather than tuning parameter values, some peatland types or regions could benefit from the implementation of more type‐ or region‐specific functions. For example, the very gentle doming of peatlands in the Cuvette Centrale complex and the slower water level recession of the in situ data (Figure 11d), both suggest that a discharge function different from what is currently implemented in PEATCLSM_Trop,Nat_ might improve model simulations over the Congo Basin. Furthermore, the elementary structure of CLSM and its input parameters was kept to allow possible integration of PEATCLSM_Trop_ in the operational GEOS CLSM framework at full spatial coverage. Including a vertical layering of the root zone (0–100 cm) with depth‐specific peat properties and a spatial diversification of the hydraulic parameters for various peatland types could, however, further improve our PEATCLSM_Trop_ modules.

PEATCLSM_Trop,Nat_ and PEATCLSM_North,Nat_ introduced a similar skill improvement compared to CLSM for natural tropical peatlands in all three regions. However, over Southeast Asia, PEATCLSM_Trop,Nat_ showed larger water level skill improvements during droughts (i.e., when the peatlands are most vulnerable), owing to the availability of extensive data from this area to constrain the model parameterization. The poor performance of both PEATCLSM_North,Nat_ and PEATCLSM_Trop,Nat_ over Central and South America, and the Congo Basin shows that peatland modules can be further improved through parameter adjustments with literature data and the implementation of new model structural changes (e.g., coupling to river stage and the effect of flooding during the wet season).

Currently, Southeast Asian peatlands are simulated with PEATCLSM_Trop_ as either all natural (PEATCLSM_Trop,Nat_) or all drained (PEATCLSM_Trop,Drain_). A drainage map that separates natural from drained peatlands over time (dynamic drainage map) would allow us to simulate only the drained peatlands with PEATCLSM_Trop,Drain_ and the natural ones with PEATCLSM_Trop,Nat_. As Bechtold et al. (2019) already suggested, a module for drained northern peatlands (PEATCLSM_North,Drain_) is needed to accurately model the role of peatlands in the global water and carbon cycles. In this research, we showed that following the same approach as for natural peatlands, a PEATCLSM_North,Drain_ module could be achieved by developing a separate parameter set for northern drained peatlands, though drainage and water management practices are very diverse (Bechtold et al., 2014).

Our spatially and temporally continuous 9‐km simulations were evaluated against water level and not against *θ*_5cm_, because in situ soil moisture data were not sufficiently available. However, remote sensing allows estimation of *θ*_5cm_, which can be linked to the water level in systems with high water levels like peatlands, where the *θ*_5cm_ and water level are strongly coupled (Bechtold et al., 2020; Dadap et al., 2019). Bechtold et al. (2020) recently showed that correlation between measured and estimated water level increased after data assimilation of Soil Moisture and Ocean Salinity (SMOS) brightness temperature (Tb) over northern peatlands using PEATCLSM_North,Nat_. Data assimilation of Tb into PEATCLSM_Trop_ could combine a specific hydrological scheme for tropical peatlands with microwave radiative transfer modeling (De Lannoy et al., 2013; Schwank et al., 2018), allowing us to develop a new data assimilation product of soil moisture and water level conditions in tropical peatlands with an unprecedented accuracy, covering all tropical peatland areas.

With the development of PEATCLSM_Trop_, we integrated peat‐specific hydrology modules for natural and drained tropical peatlands into a global LSM for the first time. These modules facilitate the integration of tropical peatland hydrology into Earth system models, possibly resulting in better understanding and projecting current and future global C fluxes (Loisel et al., 2021; Müller & Joos, 2021). Peatland hydrology and C dynamics are intrinsically linked, including in tropical peatlands where water level dynamics are the main force driving long‐term peat C sequestration (Cobb et al., 2017; Couwenberg et al., 2010; Dargie et al., 2017). A survey of 44 peat experts conducted by Loisel et al. (2021) found that the increasing uncertainty in the peat C dynamics for the future is partly due to the lack of models that estimate the effect of (changing) critical drivers, such as the water level. These PEATCLSM_Trop_ modules offer a first step towards reducing this uncertainty, and can establish a better understanding of how tropical peatlands might respond to a changing climate.

## Data Availability

Groundwater level and eddy covariance data used for evaluation are available at the sources indicated in Table B1. Full simulation output is accessible on a Zenodo data repository (Apers et al., 2022). The GEOS source code is available at https://github.com/GEOS-ESM/ and the experimental tropical PEATCLSM modules at https://github.com/mbechtold/PEATCLSM_T.

## Appendix A Propagation of Parameter Uncertainty in the Dupuit‐Forchheimer Equation Using Monte Carlo Simulations

The PEATCLSM_Trop,Drain_ *Q* function was derived from the Dupuit‐Forchheimer function of Gong et al. (2012), and uses four drainage‐related parameters. These parameters have strong variability, impacting the *Q*, and therefore, a Monte Carlo analysis of 10^5^ simulations was conducted with distributions for three of the four parameters. A normal distribution (Figure A1a) was fitted to 73 *z*_ditch_ values (Biancalani & Avagyan, 2014; Carlson et al., 2015; Evans et al., 2019; Hooijer et al., 2006; Ritzema et al., 1998; Wösten et al., 2008) obtained from measurements in acacia plantations, rubber plantations, oil palm plantations, and intensively logged forests. Figure A1b shows the *L*_ditch_ Weibull distribution that was fitted to 162 *L*_ditch_ measurements from regions that were manually labeled by Dadap et al. (2021). The *w*_strip_ is inversely related to the *L*_ditch_, therefore in each simulation the value of *w*_strip_ was directly derived from the *L*_ditch_value.

## Appendix B Overview of the Evaluation Sites and Skill Metrics

Tables B1 and B2

**Table B1:** Information on Peatland Sites (Drainage‐Based Separation, and Alphabetically Sorted by Country Initials) That Were Used for Water Level and Eddy Covariance‐Derived Evapotranspiration (ET) Evaluation

Site ID	Site location	Lon (°)	Lat (°)	Drained or undrained	# Water level and period	# ET and period	Data source	Land cover
CO_Bondoki_avg*	Bondoki; Cuvette Centrale	17.019 6	0.855 3	U	3 (2013–2014)	0	Dargie et al. (2017)	Peat swamp forest (palm dominated)
CO_Bondzale_avg*	Bondzale; Cuvette Centrale	17.977 7	1.907 0	U	3 (2013–2014)	0	Dargie et al. (2017)	Peat swamp forest (hardwood dominated)
CO_Ekolongouma_avg*	Ekolongouma; Cuvette Centrale	17.813 9	1.184 6	U	4 (2013–2018)	0	Dargie et al. (2017)	Peat swamp forest (palm dominated)
CO_Itanga_avg*	Itanga; Cuvette Centrale	17.478 2	1.212 9	U	3 (2013–2018)	0	Dargie et al. (2017)	Peat swamp forest (hardwood dominated)
IN_BR_mdm_trail_10	Mendaram; Brunei Darussalam	114.355 0	4.376 0	U	1 (2012–2013)	0	Cobb et al. (2017)	(Pristine) peat swamp forest
IN_BR_mdm_trail_6	Mendaram; Brunei Darussalam	114.354 0	4.365 0	U	2 (2012–2013)	0	Cobb et al. (2017)	(Pristine) peat swamp forest
IN_BR_mdm_trail_7	Mendaram; Brunei Darussalam	114.354 0	4.369 0	U	3 (2012–2013)	0	Cobb et al. (2017)	(Pristine) peat swamp forest
IN_BR_mdm_trail_8	Mendaram; Brunei Darussalam	114.355 0	4.371 0	U	4 (2012–2013)	0	Cobb et al. (2017)	(Pristine) peat swamp forest
IN_BRG_140312_02	Sadar Jaya; Riau	102.039 0	1.114 0	U	1 (2019–2020)	0	SIPALAGA	Information not available
IN_BRG_140412_02	Sungai Guang; Riau	103.132 0	−0.088 0	U	1 (2019–2020)	0	SIPALAGA	Information not available
IN_BRG_140806_01	Dayun; Riau	102.032 0	0.644 0	U	1 (2019–2020)	0	SIPALAGA	Information not available
IN_BRG_160205_01	Kedaton; South Sumatra	104.880 0	−3.416 0	U	1 (2019)	0	SIPALAGA	Information not available
IN_BRG_160224_02	Cinta Jaya; South Sumatra	104.965 0	−3.479 0	U	1 (2019–2020)	0	SIPALAGA	Information not available
IN_BRG_160611_01	Karang Agung; South Sumatra	104.411 0	−2.282 0	U	1 (2019–2020)	0	SIPALAGA	Information not available
IN_BRG_621101_02	Dandang; Central Kalimantan	114.081 0	−3.123 0	U	1 (2019–2020)	0	SIPALAGA	Information not available
IN_BRG_621103_04	Bukit Rawi; Central Kalimantan	113.976 0	−2.103 0	U	1 (2019–2020)	0	SIPALAGA	Information not available
IN_BRG_621107_02	Saka Kajang; Central Kalimantan	114.181 0	−2.556 0	U	1 (2019–2020)	0	SIPALAGA	Information not available
IN_BRG_621107_03	Garung; Central Kalimantan	114.221 0	−2.654 0	U	1 (2019–2020)	0	SIPALAGA	Information not available
IN_BRG_621107_04	Pilang; Central Kalimantan	114.172 0	−2.436 0	U	1 (2019)	0	SIPALAGA	Information not available
IN_BRG_630805_01	Pinang Habang; South Kalimantan	115.264 0	−2.505 0	U	1 (2019)	0	SIPALAGA	Information not available
IN_BRG16	Pulau Damar; South Kalimantan	115.369 0	−2.440 0	U	1 (2019–2020)	0	SIPALAGA	Information not available
IN_Damitdome	Damit; Brunei Darrusalam	114.363 0	4.405 0	U	1 (2012)	0	Hoyt et al. (2019)	Undrained; previously logged peat swamp forest
IN_Mendaramdome	Mendaram; Brunei Darussalam	114.352 2	4.359 9	U	1 (2013–2014)	0	Hoyt et al. (2019)	(Pristine) peat swamp forest
IN_DB_Peatland	Palangkaraya; Central Kalimantan	114.029 7	−2.338 1	U	1 (2004–2007)	0	Jauhiainen et al. (2008)	Previously deforested and drained peatland; now canal blocking and ferns as main vegetation cover
IN_SebangForest_K	Sebangau; Central Kalimantan	113.895 3	−2.321 4	U	1 (2013)	0	Könönen et al. (2016)	Logged peat swamp forest
IN_SebangRestored_L	Sebangau; Central Kalimantan	114.018 1	−2.321 7	U	1 (2012–2013)	0	Lampela et al. (2017)	Previously deforested and drained peatland; now canal blocking and ferns as main vegetation cover
IN_Sebangau_IJ‐1	Tumbang Nusa ‐ Sebangau; Central Kalimantan	114.091 3	−2.353 3	U	1 (2015–2018)	0	SATREPS ()	Peat swamp forest
IN_Sebangau_IJ‐2	Paduran Sebangau; Central Kalimantan	114.023 0	−2.573 0	U	1 (2015–2019)	0	SATREPS	Peat swamp forest
IN_Taka1_Palangkraya	Taruna Jaya; Central Kalimantan	114.059 6	−2.317 4	U	1 (2012–2019)	0	SATREPS	Previously deforested and drained peatland; now canal blocking and regenerating peatland
IN_Taka5_Sebangau	Sebangau; Central Kalimantan	114.058 1	−2.319 6	U	1 (2015–2019)	0	SATREPS	Peat swamp forest
IN_Taruna‐B1	Taruna Jaya; Central Kalimantan	114.069 5	−2.321 4	U	1 (2013–2019)	0	SATREPS	Young forest peatland
IN_AirHitam	Air Hitam; Jambi	104.116 0	−1.497 0	U	1 (2003–2004)	0	Taufik et al. (2019)	Peat swamp forest
IN_UpperSebangau_PSF	Sebangau; Central Kalimantan	114.100 0	−2.420 0	U	1 (2000–2008)	0	Taufik et al. (2019)	Peat swamp forest with minor influence of old canals
IN_Undrained_PSF	Palangkaraya; Central Kalimantan	113.908 0	−2.322 0	U	1 (2004–2007)	1 (2004–2007)	Hirano et al. (2015)	Peat swamp forest with minor influence of old canals
PA_Dipwell_avg*	Changuinola; Bocas del Toro	−82.366 0	9.382 0	U	10 (2014)	0	Baird et al. (2017)	Coastal peat swamp forest
PE_QT‐2010‐1	Quistococha; Iquitos	−73.318 9	−3.834 0	U	1 (2011–2012)	1 (2018–2019)	Lawson et al. (2014); Ameriflux	Palm dominated peat swamp‐lake complex
PE_SAM_01	Samiria; Parinari	−74.392 7	−4.835 1	U	1 (2018–2019)	0	Unpublished	Peat swamp forest (Aguajal palm dominated)
PE_SRQ_01	San Roque; Parinari	−74.629 6	−4.532 2	U	1 (2018–2019)	0	Unpublished	Peat swamp forest (Aguajal palm dominated)
PE_VEN_02_avg*	Veinte De Enero; Nauta	−73.819 3	−4.672 3	U	3 (2018–2019)	0	Unpublished	peat swamp forest (Aguajal palm dominated)
IN_BRG_140103_01	Rimba Panjang; Riau	101.299 0	0.435 0	D	1 (2019–2020)	0	SIPALAGA	Information not available
IN_BRG_140302_02	Muntai; Riau	102.433 0	1.517 0	D	1 (2019–2020)	0	SIPALAGA	Information not available
IN_BRG_140402_01	Bagan Jaya; Riau	102.987 0	−0.569 0	D	1 (2019–2020)	0	SIPALAGA	Information not available
IN_BRG_140405_02	Harapan Jaya; Riau	102.791 0	−0.477 0	D	1 (2019–2020)	0	SIPALAGA	Information not available
IN_BRG_140411_01	Kuala Sebatu; Riau	102.985 0	−0.303 0	D	1 (2019–2020)	0	SIPALAGA	Information not available
IN_BRG_140411_02	Kuala Sebatu; Riau	102.978 0	−0.372 0	D	1 (2019–2020)	0	SIPALAGA	Information not available
IN_BRG_140412_01	Sungai Gaung; Riau	103.133 0	−0.082 0	D	1 (2019–2020)	0	SIPALAGA	Information not available
IN_BRG_140508_02	Merbau; Riau	102.229 0	0.242 0	D	1 (2019–2020)	0	SIPALAGA	Information not available
IN_BRG_140508_03	Petani; Riau	102.178 0	0.306 0	D	1 (2019–2020)	0	SIPALAGA	Information not available
IN_BRG_140509_01	Kuala Panduk; Riau	102.335 0	0.186 0	D	1 (2019–2020)	0	SIPALAGA	Information not available
IN_BRG_140801_01	Kampung Rempak; Riau	102.001 0	0.826 0	D	1 (2019–2020)	0	SIPALAGA	Information not available
IN_BRG_140802_02	Penyengat; Riau	102.354 0	0.831 0	D	1 (2019–2020)	0	SIPALAGA	Information not available
IN_BRG_140810_01	Sam Sam; Riau	101.072 0	0.966 0	D	1 (2019–2020)	0	SIPALAGA	Information not available
IN_BRG_141006_01	Semukut; Riau	102.551 0	1.012 0	D	1 (2019–2020)	0	SIPALAGA	Information not available
IN_BRG_147204_01	Bangsal Aceh; Riau	101.297 0	1.628 0	D	1 (2019–2020)	0	SIPALAGA	Information not available
IN_BRG_147205_01	Pelintung; Riau	101.646 0	1.614 0	D	1 (2019–2020)	0	SIPALAGA	Information not available
IN_BRG_147205_02	Teluk Makmur; Riau	101.545 0	1.626 0	D	1 (2019–2020)	0	SIPALAGA	Information not available
IN_BRG_150611_01	Bram Itam Kanan; Jambi	103.321 0	−0.908 0	D	1 (2019–2020)	0	SIPALAGA	Information not available
IN_BRG_150611_02	Bram Itam Kanan; Jambi	103.355 0	−0.922 0	D	1 (2019–2020)	0	SIPALAGA	Information not available
IN_BRG_150710_01	Sungai Beras; Jambi	103.680 0	−1.232 0	D	1 (2019–2020)	0	SIPALAGA	Information not available
IN_BRG_150710_03	Pandan Sajahtera; Jambi	103.773 0	−1.298 0	D	1 (2019–2020)	0	SIPALAGA	Information not available
IN_BRG_160224_01	Cinta Jaya; South Sumatra	104.977 0	−3.392 0	D	1 (2019–2020)	0	SIPALAGA	Information not available
IN_BRG_160224_03	Cinta Jaya; South Sumatra	104.965 0	−3.432 0	D	1 (2019–2020)	0	SIPALAGA	Information not available
IN_BRG_160609_01	Muara Medak; South Sumatra	103.929 0	−1.795 0	D	1 (2019–2020)	0	SIPALAGA	Information not available
IN_BRG_610102_02	Berlimang; West Kalimantan	109.178 0	1.379 0	D	1 (2019–2020)	0	SIPALAGA	Information not available
IN_BRG_610117_01	Semata; West Kalimantan	109.145 0	1.515 0	D	1 (2019–2020)	0	SIPALAGA	Information not available
IN_BRG_610208_01	Antibar; West Kalimantan	109.262 0	0.115 0	D	1 (2019–2020)	0	SIPALAGA	Information not available
IN_BRG_610218_01	Anjungan Dalam; West Kalimantan	109.020 0	0.385 0	D	1 (2019–2020)	0	SIPALAGA	Information not available
IN_BRG_611202_01	Simpang Kanan; West Kalimantan	109.423 0	0.111 0	D	1 (2020)	0	SIPALAGA	Information not available
IN_BRG_611203_02	Simpang Kanan; West Kalimantan	109.476 0	−0.088 0	D	1 (2019–2020)	0	SIPALAGA	Information not available
IN_BRG_611206_01	Olak Olak; West Kalimantan	109.365 0	−0.491 0	D	1 (2019–2020)	0	SIPALAGA	Information not available
IN_BRG_611209_01	Punggur Kecil; West Kalimantan	109.328 0	−0.129 0	D	1 (2019–2020)	0	SIPALAGA	Information not available
IN_BRG_620309_02	Pulang Pisau; Central Kalimantan	114.400 0	−2.538 0	D	1 (2019–2020)	0	SIPALAGA	Information not available
IN_BRG_621103_03	Sigi; Central Kalimantan	113.959 0	−2.032 0	D	1 (2019–2020)	0	SIPALAGA	Information not available
IN_BRG_621105_02	Kalawa; Central Kalimantan	114.220 0	−2.711 0	D	1 (2019)	0	SIPALAGA	Information not available
IN_BRG_621105_03	Buntoi; Central Kalamintan	114.175 0	−2.838 0	D	1 (2019–2020)	0	SIPALAGA	Information not available
IN_BRG_621107_06	Jabiren; Central Kalimantan	114.169 0	−2.549 0	D	1 (2019–2020)	0	SIPALAGA	Information not available
IN_BRG_621108_01	Medura Sebangau; Central Kalimantan	113.763 0	−2.903 0	D	1 (2019–2020)	0	SIPALAGA	Information not available
IN_BRG_627104_04	Kereng Bangkirai; Central Kalimantan	113.880 0	−2.287 0	D	1 (2019)	0	SIPALAGA	Information not available
IN_BRG_630708_01	Haur Gading; South Kalimantan	115.401 0	−2.462 0	D	1 (2019–2020)	0	SIPALAGA	Information not available
IN_BRG_631104_01	Mantimin; South Kalimantan	115.386 0	−2.397 0	D	1 (2019–2020)	0	SIPALAGA	Information not available
IN_BRG_910111_01	Sumber Mulya; Papua	140.216 0	−8.205 0	D	1 (2019–2020)	0	SIPALAGA	Information not available
IN_BRG11	Anjir Kalampan; Central Kalimantan	114.313 0	−2.819 0	D	1 (2019–2020)	0	SIPALAGA	Information not available
IN_BRG12	Katunjung; Central Kalimantan	114.464 0	−2.239 0	D	1 (2019)	0	SIPALAGA	Information not available
IN_BRG3	Sumber Agung; Jambi	103.882 0	−1.711 0	D	1 (2019–2020)	0	SIPALAGA	Information not available
IN_BRG5	Gedong Karya; Jambi	104.026 0	−1.382 0	D	1 (2019–2020)	0	SIPALAGA	Information not available
IN_Drained_PSF	Palangkaraya; Central Kalimantan	114.036 0	−2.346 0	D	1 (2004–2007)	1 (2004–2007)	Hirano et al. (2015)	Drained and previously logged secondary peat swamp forest
IN_Jambi1	Tanjung Jabung Timur; Jambi	103.590 0	−1.238 0	D	1 (2016–2019)	0	SATREPS	Drained peat swamp forest
IN_DF_Peatland	Palangkaraya; Central Kalimantan	114.036 7	−2.345 0	D	1 (2004–2007)	0	Jauhiainen et al. (2008)	Drained peat swamp forest
IN_Kalbar1	Kalbar; West Kalimantan	109.395 0	−0.210 0	D	1 (2016–2020)	0	SATREPS	Small‐holder agriculture (including oil palm plantations)
IN_Kalteng1	Kalteng; Central Kalimantan	114.058 0	−2.320 0	D	1 (2016–2020)	0	SATREPS	Drained and cleared area between two peat swamp forests
IN_N_Selangor	Raja Musa; North Selangor	101.306 7	3.425 6	D	1 (2018–2019)	0	Unpublished	Drained small‐holder agriculture (mainly second or third rotation oil palm on shallow peat)
IN_Palangkaraya	Hampangen; Central Kalimantan	113.578 7	−1.920 0	D	1 (2018–2019)	0	Unpublished	Drained small‐holder agriculture (including oil palm and rubber tree plantations)
IN_Pontianak	Teluk Empening; West Kalimantan	109.591 4	−0.380 7	D	1 (2018–2019)	0	Unpublished	Drained small‐holder agriculture (mainly ginger and rubber tree plantations on shallow peat)
IN_Riau1	Tanjung Leban; Riau	101.737 2	1.642 4	D	1 (2016–2017)	0	SATREPS	Drained peat swamp forest
IN_Taka4	Palangkaraya; Central Kalimantan	114.572 4	−2.578 1	D	1 (2012–2014)	0	SATREPS	Oil palm plantation
IN_Taka7	Pontianak; West Kalimantan	109.697 1	0.005 2	D	1 (2013–2015)	0	SATREPS	Oil palm plantation

*Note.* All sites of the same peatland complex were aggregated and marked with “_avg*”; Lat: latitude; Lon: longitude; # WT: number of water level monitoring wells; # EC: number of eddy covariance sites.

**Table B2:** Skill Metrics for Water Level and ET Measurements at the 96 Evaluation Sites

Site ID	Bias (m)		RMSD (m)		ubRMSD (m)		R (−)		anomR (−)	
	CLSM	PEATCLSM_Trop_	CLSM	PEATCLSM_Trop_	CLSM	PEATCLSM_Trop_	CLSM	PEATCLSM_Trop_	CLSM	PEATCLSM_Trop_
CO_Bondoki_avg*	−4.36	−0.50	4.37	0.54	0.31	0.20	0.78	0.77	‐	‐
CO_Bondzale_avg*	−2.26	−0.45	2.36	0.57	0.67	0.35	0.40	0.31	‐	‐
CO_Ekolongouma_avg*	−2.13	−0.28	2.24	0.43	0.70	0.32	0.22	0.33	−0.23	−0.11
CO_Itanga_avg*	−3.62	−0.26	3.69	0.40	0.73	0.31	0.21	0.47	−0.13	0.19
IN_BR_mdm_trail_10	0.02	0.04	0.47	0.09	0.47	0.08	0.72	0.71	‐	‐
IN_BR_mdm_trail_6	0.02	0.04	0.46	0.09	0.46	0.08	0.81	0.63	‐	‐
IN_BR_mdm_trail_7	0.02	0.04	0.47	0.08	0.47	0.07	0.79	0.65	‐	‐
IN_BR_mdm_trail_8	0.02	0.04	0.47	0.08	0.47	0.08	0.78	0.68	‐	‐
IN_BRG_140312_02	−1.17	0.17	1.25	0.26	0.46	0.20	0.45	0.70	‐	‐
IN_BRG_140412_02	−1.36	0.16	1.59	0.25	0.81	0.19	0.51	0.74	‐	‐
IN_BRG_140806_01	−1.35	0.10	1.44	0.18	0.51	0.15	0.66	0.84	‐	‐
IN_BRG_160205_01	−1.66	−0.16	1.93	0.22	0.98	0.16	0.98	0.98	‐	‐
IN_BRG_160224_02	−0.88	0.14	1.66	0.33	1.40	0.29	0.73	0.72	‐	‐
IN_BRG_160611_01	−0.78	0.14	1.51	0.20	1.29	0.14	0.78	0.93	‐	‐
IN_BRG_621101_02	−0.75	−0.15	1.92	0.36	1.77	0.32	0.67	0.75	‐	‐
IN_BRG_621103_04	−0.42	−0.04	1.33	0.20	1.27	0.20	0.75	0.86	‐	‐
IN_BRG_621107_02	−0.88	−0.04	1.74	0.25	1.50	0.25	0.70	0.84	‐	‐
IN_BRG_621107_03	−0.49	0.05	1.50	0.21	1.42	0.21	0.75	0.90	‐	‐
IN_BRG_621107_04	0.27	0.16	0.51	0.21	0.43	0.14	0.92	0.79	‐	‐
IN_BRG_630805_01	−0.11	0.18	0.83	0.25	0.82	0.17	0.94	0.94	‐	‐
IN_BRG16	−0.26	0.04	1.12	0.15	1.09	0.14	0.77	0.97	‐	‐
IN_Damitdome	−0.19	0.05	0.45	0.12	0.41	0.10	0.85	0.70	‐	‐
IN_Mendaramdome	0.65	−0.15	1.00	0.19	0.76	0.12	0.92	0.92	‐	‐
IN_DB_Peatland	−0.75	−0.02	1.38	0.20	1.16	0.20	0.64	0.83	0.61	0.74
IN_SebangForest_K	−0.40	−0.04	1.03	0.16	0.95	0.16	0.77	0.80	‐	‐
IN_SebangRestored_L	0.13	0.03	0.75	0.12	0.74	0.11	0.67	0.71	‐	‐
IN_Sebangau_IJ‐1	−0.13	0.00	1.08	0.14	1.07	0.14	0.75	0.89	‐	‐
IN_Sebangau_IJ‐2	−1.03	−0.18	1.56	0.28	1.17	0.21	0.86	0.84	‐	‐
IN_Taka1_Palangkraya	−0.07	0.04	0.99	0.25	0.99	0.24	0.62	0.66	0.54	0.54
IN_Taka5_Sebangau	−0.20	−0.03	1.08	0.15	1.06	0.14	0.84	0.92	0.77	0.90
IN_Taruna‐B1	−0.05	0.10	1.03	0.23	1.03	0.20	0.71	0.77	0.64	0.69
IN_AirHitam	−0.73	0.06	1.16	0.13	0.91	0.11	0.52	0.86	‐	‐
IN_UpperSebangau_PSF	−0.79	−0.01	1.37	0.18	1.11	0.18	0.74	0.88	0.40	0.61
IN_Undrained_PSF	−0.60	0.01	1.22	0.12	1.06	0.12	0.86	0.94	0.76	0.88
PA_Dipwell_avg*	0.12	0.04	0.14	0.05	0.06	0.03	0.15	0.07	‐	‐
PE_QT‐2010‐1	−2.14	−0.30	2.49	0.44	1.29	0.32	0.66	0.53	‐	‐
PE_SAM_01	−0.48	−0.28	0.96	0.44	0.83	0.34	0.38	0.45	‐	‐
PE_SRQ_01	−1.09	−0.11	1.11	0.21	0.22	0.18	0.48	0.62	‐	‐
PE_VEN_02_avg*	0.15	−0.04	0.80	0.14	0.79	0.14	0.34	0.43	‐	‐
IN_BRG_140103_01	−0.86	−0.14	1.05	0.32	0.60	0.28	0.38	0.78	‐	‐
IN_BRG_140302_02	−1.04	−0.28	1.25	0.47	0.70	0.37	0.08	0.45	‐	‐
IN_BRG_140402_01	−0.62	−0.03	1.07	0.31	0.87	0.31	0.52	0.72	‐	‐
IN_BRG_140405_02	0.10	0.00	0.75	0.26	0.74	0.26	0.81	0.82	‐	‐
IN_BRG_140411_01	−0.48	0.02	0.95	0.26	0.82	0.26	0.39	0.49	‐	‐
IN_BRG_140411_02	−0.10	0.33	0.85	0.41	0.84	0.24	0.50	0.77	‐	‐
IN_BRG_140412_01	−0.84	−0.09	1.23	0.37	0.91	0.36	0.52	0.75	‐	‐
IN_BRG_140508_02	−0.61	−0.29	0.91	0.36	0.67	0.22	0.48	0.70	‐	‐
IN_BRG_140508_03	−0.49	−0.12	0.70	0.23	0.50	0.20	0.57	0.79	‐	‐
IN_BRG_140509_01	0.31	0.36	0.69	0.42	0.61	0.22	0.63	0.73	‐	‐
IN_BRG_140801_01	−0.64	0.07	0.97	0.38	0.72	0.37	0.16	0.44	‐	‐
IN_BRG_140802_02	−1.15	−0.46	1.31	0.54	0.61	0.27	0.49	0.66	‐	‐
IN_BRG_140810_01	−0.20	−0.07	0.67	0.28	0.64	0.27	0.08	0.27	‐	‐
IN_BRG_141006_01	−1.41	−0.46	1.49	0.52	0.49	0.25	0.51	0.82	‐	‐
IN_BRG_147204_01	−1.00	−0.33	1.23	0.45	0.71	0.31	0.55	0.69	‐	‐
IN_BRG_147205_01	−0.53	−0.34	0.85	0.46	0.67	0.32	0.02	0.35	‐	‐
IN_BRG_147205_02	−1.03	−0.40	1.23	0.53	0.66	0.34	−0.08	0.27	‐	‐
IN_BRG_150611_01	−0.15	0.15	0.99	0.36	0.98	0.33	0.56	0.74	‐	‐
IN_BRG_150611_02	−0.57	−0.39	1.12	0.48	0.96	0.28	0.78	0.83	‐	‐
IN_BRG_150710_01	−0.40	−0.51	1.13	0.61	1.06	0.35	0.71	0.78	‐	‐
IN_BRG_150710_03	−0.92	−0.63	1.48	0.74	1.16	0.38	0.42	0.70	‐	‐
IN_BRG_160224_01	−0.83	−0.41	1.58	0.53	1.35	0.33	0.82	0.87	‐	‐
IN_BRG_160224_03	−1.40	−0.34	1.83	0.51	1.18	0.38	0.95	0.84	‐	‐
IN_BRG_160609_01	−0.26	−0.32	1.14	0.42	1.11	0.27	0.66	0.86	‐	‐
IN_BRG_610102_02	0.58	0.33	0.98	0.39	0.79	0.21	0.46	0.56	‐	‐
IN_BRG_610117_01	−0.06	−0.14	0.63	0.20	0.63	0.14	0.76	0.89	‐	‐
IN_BRG_610208_01	0.99	0.10	1.17	0.16	0.62	0.13	0.70	0.77	‐	‐
IN_BRG_610218_01	0.49	0.19	0.92	0.28	0.77	0.20	0.46	0.60	‐	‐
IN_BRG_611202_01	1.03	0.19	1.08	0.21	0.31	0.09	0.32	0.46	‐	‐
IN_BRG_611203_02	0.82	−0.02	1.04	0.16	0.65	0.16	0.74	0.80	‐	‐
IN_BRG_611206_01	0.83	0.09	1.14	0.22	0.78	0.20	0.69	0.70	‐	‐
IN_BRG_611209_01	0.64	−0.11	0.91	0.18	0.65	0.14	0.80	0.87	‐	‐
IN_BRG_620309_02	−0.85	−0.58	2.09	0.81	1.91	0.57	0.55	0.70	‐	‐
IN_BRG_621103_03	−0.13	−0.33	1.31	0.54	1.30	0.43	0.69	0.73	‐	‐
IN_BRG_621105_02	−0.67	−0.46	1.42	0.53	1.25	0.26	0.95	0.97	‐	‐
IN_BRG_621105_03	0.19	0.04	1.19	0.28	1.17	0.28	0.88	0.89	‐	‐
IN_BRG_621107_06	−0.20	−0.32	1.34	0.47	1.33	0.34	0.74	0.86	‐	‐
IN_BRG_621108_01	−0.37	−0.36	1.61	0.58	1.56	0.46	0.65	0.74	‐	‐
IN_BRG_627104_04	0.51	−0.13	0.60	0.19	0.32	0.14	0.95	0.88	‐	‐
IN_BRG_630708_01	0.61	0.07	1.46	0.28	1.33	0.27	0.83	0.94	‐	‐
IN_BRG_631104_01	0.45	−0.18	1.20	0.52	1.12	0.49	0.49	0.53	‐	‐
IN_BRG_910111_01	−3.23	−0.65	3.35	0.76	0.87	0.41	0.55	0.78	‐	‐
IN_BRG11	0.75	0.56	1.32	0.67	1.09	0.38	0.83	0.76	‐	‐
IN_BRG12	0.46	−0.08	0.84	0.22	0.70	0.20	0.89	0.86	‐	‐
IN_BRG3	0.30	0.13	1.08	0.28	1.04	0.25	0.15	0.57	‐	‐
IN_BRG5	0.05	−0.29	1.08	0.38	1.08	0.25	0.79	0.92	‐	‐
IN_Drained_PSF	−0.30	−0.21	1.15	0.33	1.11	0.25	0.77	0.91	0.64	0.79
IN_Jambi1	0.35	−0.15	0.69	0.21	0.59	0.15	0.80	0.82	0.34	0.48
IN_DF_Peatland	−0.44	−0.20	1.15	0.33	1.06	0.26	0.71	0.84	‐	‐
IN_Kalbar1	0.89	0.10	1.09	0.20	0.63	0.17	0.56	0.68	0.48	0.59
IN_Kalteng1	0.19	−0.25	0.98	0.33	0.96	0.22	0.73	0.86	0.74	0.84
IN_N_Selangor	0.25	−0.02	0.62	0.14	0.57	0.14	0.83	0.81	‐	‐
IN_Palangkaraya	−0.63	−0.48	1.31	0.65	1.15	0.45	0.33	0.52	‐	‐
IN_Pontianak	0.87	−0.02	1.13	0.22	0.73	0.22	0.62	0.80	‐	‐
IN_Riau1	0.59	−0.06	0.69	0.15	0.37	0.13	0.32	0.39	‐	‐
IN_Taka4	−0.05	−0.20	1.02	0.36	1.02	0.30	0.51	0.64	‐	‐
IN_Taka7	0.61	−0.11	0.83	0.23	0.56	0.20	0.55	0.55	‐	‐

*Note.* All sites of the same peatland complex were aggregated and marked with “_avg*”.
